# Immune activation and HIV-specific T cell responses are modulated by a cyclooxygenase-2 inhibitor in untreated HIV-infected individuals: An exploratory clinical trial

**DOI:** 10.1371/journal.pone.0176527

**Published:** 2017-05-02

**Authors:** Christian Prebensen, Marius Trøseid, Thor Ueland, Anders Dahm, Per Morten Sandset, Ingeborg Aaberge, Kristian Waalen, Anne Ma Dyrhol-Riise, Kjetil Taskén, Dag Kvale

**Affiliations:** 1 Department of Infectious Diseases, Oslo University Hospital, Oslo, Norway; 2 Institute of Clinical Medicine, University of Oslo, Oslo, Norway; 3 KG Jebsen Inflammation Research Center, University of Oslo, Oslo, Norway; 4 Research Institute of Internal Medicine, Oslo University Hospital, Oslo, Norway; 5 Center of Hematological Research, Akershus University Hospital, Oslo, Norway; 6 Department of Hematology, Oslo University Hospital, Oslo, Norway; 7 Norwegian Institute of Public Health, Oslo, Norway; 8 Department of Clinical Science, University of Bergen, Bergen, Norway; 9 Centre for Molecular Medicine Norway, University of Oslo, Oslo, Norway; Rush University, UNITED STATES

## Abstract

Pathologically elevated immune activation and inflammation contribute to HIV disease progression and immunodeficiency, potentially mediated by elevated levels of prostaglandin E_2_, which suppress HIV-specific T cell responses. We have previously shown that a high dose of the cyclooxygenase-2 inhibitor celecoxib can reduce HIV-associated immune activation and improve IgG responses to T cell-dependent vaccines. In this follow-up study, we included 56 HIV-infected adults, 28 antiretroviral therapy (ART)-naïve and 28 on ART with undetectable plasma viremia but CD4 counts below 500 cells/μL. Patients in each of the two study groups were randomized to receive 90 mg qd of the cyclooxygenase-2 inhibitor etoricoxib for six months, two weeks or to a control arm, respectively. T cell activation status, HIV Gag-specific T cell responses and plasma inflammatory markers, tryptophan metabolism and thrombin generation were analyzed at baseline and after four months. In addition, patients received tetanus toxoid, conjugated pneumococcal and seasonal influenza vaccines, to which IgG responses were determined after four weeks. In ART-naïve patients, etoricoxib reduced the density of the activation marker CD38 in multiple CD8^+^ T cell subsets, improved Gag-specific T cell responses, and reduced *in vitro* plasma thrombin generation, while no effects were seen on plasma markers of inflammation or tryptophan metabolism. No significant immunological effects of etoricoxib were observed in ART-treated patients. Patients receiving long-term etoricoxib treatment had poorer tetanus toxoid and conjugated pneumococcal vaccine responses than those receiving short-course etoricoxib. Cyclooxygenase-2 inhibitors may attenuate harmful immune activation in HIV-infected patients without access to ART.

## Introduction

Chronic, untreated HIV infection is characterized by a state of pathological immune activation and inflammation, which contributes to disease progression and immunodeficiency [1]. Expression of the activation marker CD38 on T cells predicts both progression to AIDS and mortality [2–6]. When plasma viremia is suppressed to near-undetectable levels by antiretroviral therapy (ART), immune activation is attenuated, but not to the level of the HIV-uninfected population [7, 8], and residual immune activation in patients on ART is associated with both mortality and impaired immune reconstitution [7, 9, 10]. Of particular concern are the effects of HIV infection on cardiovascular health, with HIV-infected patients at an increased risk of both myocardial infarction and venous thromboembolism [11, 12].

In the absence of ART, HIV-specific CD8^+^ T cell function is essential for viral control [13, 14], but this is progressively lost in most chronically infected patients [15] and not restored after ART initiation [16, 17]. In recent years, there has been an increasing focus on treatment strategies to induce viral control post-ART, a so-called “functional cure” [18]. Most cure strategies will likely rely on boosting HIV-specific CD8^+^ T cell function to eliminate the majority of latently infected cells and prevent viral rebound from any remaining reservoirs [19, 20].

A major driver of HIV immunopathogenesis is the translocation of microbial products from the gut lumen to the submucosa and circulation, due to a defective gut barrier [21]. This chronic exposure to microbial antigens such as lipopolysaccharide (LPS) activates innate immune cells, including monocytes, macrophages and dendritic cells, inducing the enzyme cyclooxygenase (COX) 2 and leading to increased synthesis of prostaglandin E_2_ (PGE_2_) [22–25]. We have hypothesized that this represents one mechanism of functional suppression of T cells in HIV infection, as PGE_2_ inhibits T cell activation via a cyclic AMP-/protein kinase A-dependent mechanism [26–28]. Another enzyme induced by LPS exposure of innate immune cells is indoleamine 2,3-dioxygenase (IDO), which catabolizes tryptophan and inhibits T cell responses in both HIV infection and cancer [29, 30].

We have previously shown that treatment with high-dose COX-2 inhibitors (COX-2i) for 12 weeks can reduce the expression of T cell activation markers in both untreated [31] and treated but viremic [32] HIV-infected patients, and improve IgG recall responses to a T cell-dependent vaccine in ART-naïve patients [31]. However, in ART-naïve patients with high levels of immune activation at baseline, celecoxib administered at twice the typically recommended maximal dose (400mg bid) was also associated with a high incidence of rash. This explorative study was performed to further characterize the immunological effects of a longer treatment course of a COX-2i at a conventional clinical dose which would be better tolerated, in both ART-naïve, viremic patients and ART-treated patients with suppressed plasma viremia but suboptimal CD4 T cell reconstitution. Patients were randomized to receive 90 mg qd of the COX-2i etoricoxib, a commonly used dose in inflammatory rheumatic disease, for six months or two weeks, or to a control group. T cell activation status, HIV Gag-specific T cell function, and plasma inflammatory markers, tryptophan metabolism and thrombin generation were examined at baseline and after four months, to determine the immunomodulatory effect of long-term COX-2i treatment. The two week treatment group was included to test the hypothesis that a short course of COX-2i could improve IgG responses to three T cell-dependent vaccines, i.e. serve as a vaccine adjuvant in HIV-infected individuals.

## Materials and methods

The CONSORT checklist and study protocol are available as supplementary information; see S1 and S2 Files.

### Patient inclusion

Adult, HIV-infected patients were included from the outpatient clinic at the Department of Infectious Diseases, Oslo University Hospital, from January 2011 through May 2014, in one of two study groups. One group consisted of ART-naïve patients, with CD4 counts above 350 cells/μL and plasma HIV RNA above 2000 copies/mL, a confirmed diagnosis of HIV infection less than 8 years pre-inclusion and no current indication for ART according to European guidelines at the time [33]. The other study group consisted of patients on ART, with CD4 counts below 500 cells/μL and HIV RNA below 50 copies/mL for at least 6 months prior to inclusion. All included patients were between 18 and 65 years of age and had no history of symptomatic acute HIV infection within the last 12 months.

Exclusion criteria for all study participants were concomitant use of non-steroidal anti-inflammatory drugs, corticosteroids or interferon-alpha, total cholesterol >7 mM, elevated serum creatinine, creatinine clearance <30ml/min, hypertension, heart failure, diabetes, ischemic heart disease, peripheral arteriosclerosis and/or cerebrovascular disease, cardiovascular events or stroke in parents, siblings or offspring of <55 years of age, pregnancy or insufficient birth-control, breast-feeding, deranged liver function, inflammatory bowel disease, active peptic ulcer, or gastrointestinal haemorrhage.

### Study design

ART-naïve and ART-treated patients were separately randomized to one of three arms in a 2:1:1 fashion and in blocks of 20, by drawing allocation numbers from a pre-generated random sequence to which the investigators were blinded beforehand. Arm 1 received oral etoricoxib 90 mg once daily for six months, arm 2 received oral etoricoxib 90 mg once daily for two weeks whereas arm 3 received no drug. The study was open label. Patients were examined and blood samples were drawn at screening, inclusion (week 0), study week 5 in arms 2/3, study week 9 in arm 1, four months and six months.

**Fig 1:**
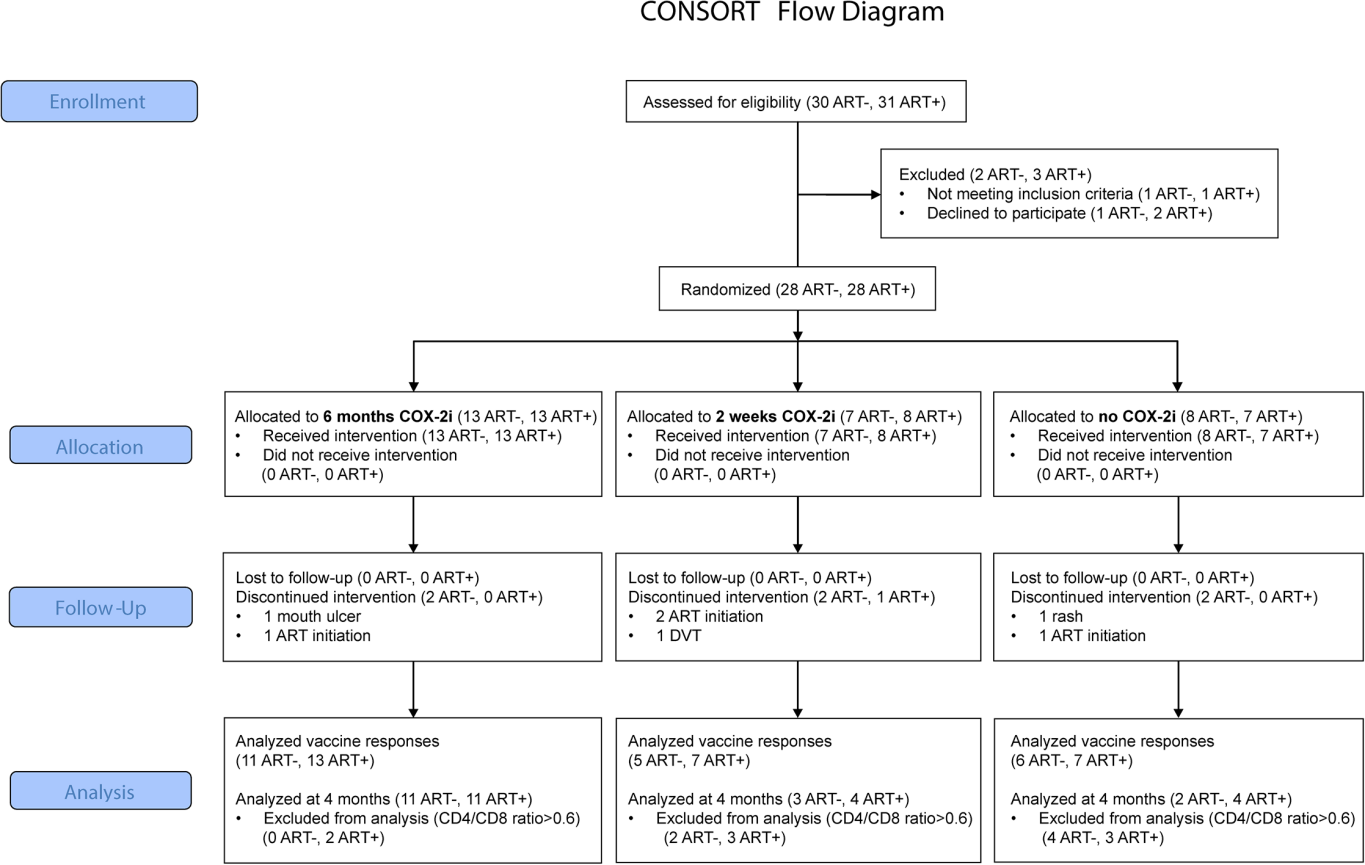
CONSORT flow chart.

**Fig 2:**
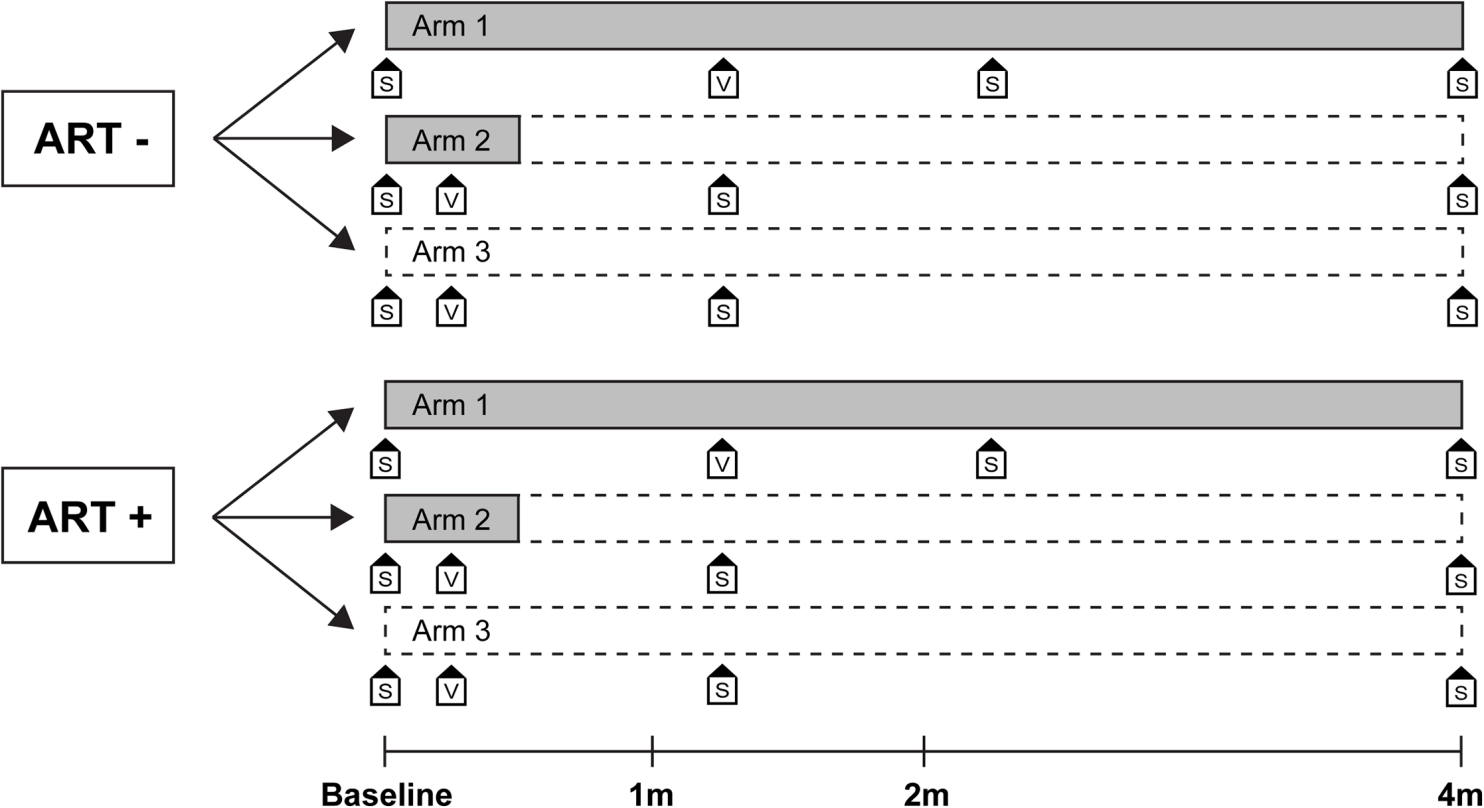
Study overview. COX-2i treatment duration indicated in grey. S, blood sampling; V, vaccination.

Final analysis of immunological endpoints was performed at four months as opposed to the protocol-ascribed six months. This was due to illness of key study personnel, leading to delayed sampling at six months of a significant proportion of patients, after cessation of study drug. Thus, endpoint data related to COX-2i effects beyond four months of follow-up were incomplete. Notably, no patients discontinued the study due to adverse events or commencement of ART after four months of follow-up (see Fig 1 for patient flow and Fig 2 for overview study design).

Written, informed consent was obtained from all study participants. The study was approved by the Norwegian Medicines Agency (EudraCT No: 2010-020382-25) and the Norwegian Regional Committee for Medical and Health Research Ethics South East (application #2010/1593). The study was registered at Clinicaltrials.gov (Identifier: NCT01269515). The study was independently monitored for safety by Link Medical.

### Endpoints

Primary endpoints were changes in CD38 density on CD8^+^ T cells after four months of etoricoxib treatment and changes in IgG levels to the three study vaccines four weeks post-vaccination. Secondary endpoints were changes in HIV Gag-induced CD8^+^ T cell responses and plasma markers of inflammation, tryptophan metabolism and coagulation after four months.

### Safety evaluation

Adverse events were registered at all study visits, and graded as non-serious/serious and likely related to etoricoxib or not. Routine clinical biochemistry parameters were analyzed at all sampling time points, and values outside reference ranges were evaluated by a clinical investigator.

### Routine laboratory analyses

CD4^+^ and CD8^+^ T cell counts and plasma HIV RNA was determined at all sampling time points by routine clinical assays.

### Flow cytometry

To characterize CD8^+^ T cell subsets, thawed peripheral blood mononuclear cells (PBMC) were surface-stained with αCD3 V450, αCD8 APC-H7, αCD38 PE, αPD-1 FITC, αCD45RA APC, αCD27 PerCP Cy-5.5 and αCCR7 PE-Cy7 (all BD Biosciences, San Jose, CA), then analyzed. To quantify regulatory T cells thawed PBMC were first incubated with Fixable Viability Stain 450 (BD), then surface-stained with αCD3 PerCP Cy-5.5, αCD4 APC-H7, αCD25 FITC, αCD45RA APC (all BD) and αCD127 PE-Cy7 (eBioscience, San Diego, CA). Subsequently, cells were fixed and permeabilized using the FoxP3 Buffer set (BD), followed by intracellular staining with αFoxP3 PE (BD).

The density of activation marker CD38 on CD8^+^ subsets was determined by the Quantibrite method [34]. The αCD38 PE monoclonal antibody (mAb) used to stain CD8^+^ T cells had >95% 1:1 PE:mAb ratio. By comparing PE MFI with that of Quantibrite beads (BD) with four known densities of PE, the number of CD38 molecules per CD8 cell was calculated.

To quantify HIV-specific CD8^+^ T cell effector responses, thawed PBMC were rested overnight and tested for viability by Muse Count and Viability Assay (Merck Millipore, Darmstadt, Germany) before stimulation with an overlapping, 15-mer HIV Gag peptide pool (PepMix HIV Gag Ultra, JPT Peptide Solutions, Berlin, Germany) at a final concentration of 2 μg/mL/peptide. Costimulatory antibodies αCD28 and αCD49d (1 μg/mL; BD), brefeldin A (GolgiPlug, 1 μL/mL; BD), monensin (GolgiStop; 0,67 μL/mL; BD), and αCD107a PE were added at the start of the stimulation. Unstimulated and positive controls (Staphylococcal enterotoxin B, 1 μg/mL; Sigma-Aldrich) were included for all samples. Cells were incubated for 5.5 hours at 37°C, incubated with Fixable Viability Stain 450, fixed and permeabilized using BD Cytofix/Cytoperm, and stained with αCD3 PerCP, αCD8 APC-H7, αIL-2 APC, αIFN-γ PE-Cy7, αMIP-1β FITC and αTNF-α BV605 (all BD).

Cells were analyzed on a BD FACS Canto II flow cytometer, data was acquired using FACS Diva 6.1 (BD) and analyzed using FlowJo X (FlowJo LLC, Ashland, OR). Gating of non-discrete populations was done using fluorescence minus-one (FMO) controls, where appropriate. Examples of all gates are included in S1 Fig.

CD8 effector responses were background-subtracted using the unstimulated control, with responses <0.01% adjusted to 0. Polyfunctional subsets were defined by Boolean gating, while a total Gag-specific response was calculated by summing all 4 cytokine responses and the CD107a response per sample. Polyfunctional response data were graphed and analyzed in SPICE (M Roederer, NIH, [35]).

### Plasma soluble markers

Soluble (s)CD25, sCD163, sCD14, interleukin (IL) 6 (high sensitivity), interferon-inducible protein (IP) 10 and C-reactive protein (CRP) were measured in cryopreserved EDTA plasma by ELISA (R&D Systems, Stillwater, MN).

### Coagulation analyses

Thrombin formation in cryopreserved citrate plasma was assayed using the CAT assay [36] according to the user manual provided by Thrombinoscope B.V (Maastricht, The Netherlands). From the CAT assay we analyzed the parameters ETP, ie. the area under the thrombin generation curve and Peak, the maximum concentration of thrombin. Coagulation was triggered by recalcification of plasma in the presence of 5 pM recombinant, relipidated human tissue factor (PPP-reagent, Thrombinoscope B.V), and 417 μM fluorogenic substrate (FluCa, Thrombinoscope B.V). Fluorescence was monitored using the Fluoroscan Ascent fluorometer (ThermoLabsystems, Helsinki, Finland), and the thrombin generation parameters were calculated using the Thrombinoscope® software (Thrombino-scope B.V). In order to increase reproducibility all CAT-derived parameters were normalized by dividing the measured test value with that of reference plasma pooled from 34 healthy adults run in the same assay.

Free protein S, free Tissue Factor Plasminogen Inhibitor (TFPI) and D-dimer were analyzed in cryopreserved citrate plasma using commercial ELISA kits (Zymutest free protein S from Hyphen Biomed, Neuville Sur Oise, France; Asserachrom® Free TFPI and Asserachrom® D-Dimer from Diagnostica Stago, Asnières, France).

### LC-MS K/T ratio

Plasma concentrations of tryptophan and its metabolite kynurenine in cryopreserved EDTA plasma were analyzed by liquid chromatography—tandem mass spectrometry by Bevital AS (Bergen, Norway) [37].

### Vaccination substudy

To investigate the effect of long-term vs. short-course etoricoxib treatment on vaccine IgG responses, study participants received the following three T cell-dependent vaccines at study week 5 (arm 1) and study week 1 (arms 2/3): seasonal influenza virus type A/B vaccine (Fluarix, GlaxoSmithKline, Brentford, UK), tetanus toxoid vaccine (Tetavax, Sanofi Pasteur MSD, Swiftwater, PA) and 13-valent pneumococcal conjugate vaccine (Prevenar 13, Pfizer, New York, NY). Participants who had received one of the study vaccines within the preceding 2 years were not re-vaccinated and were excluded from analysis of responses to the vaccine in question. Vaccine-specific IgG levels were measured in cryopreserved sera at baseline and 4 weeks after vaccination (study week 9 for arm 1, study week 5 for arms 2/3).

IgG antibodies to tetanus toxoid were quantified by ELISA. Sera were tested at two-fold dilutions, and antibody levels were determined according to the International Standard for Tetanus Immunoglobulin (Human WHO TE-3, NIBSC, Hertfordshire, UK). IgG antibody levels above 0.1 IU/mL were considered protective. All sera from a given patient were run on the same plate.

Total IgG antibodies to a mix of 23 pneumococcal polysaccharides (PPV23, Pneumovax, Sanofi Pasteur MSD), were measured by ELISA after Cell Wall Polysaccharide (CWPS) adsorption of sera (PnC C-PS Pneumococcal C polysaccharide, Statens Serum Institut, Copenhagen, Denmark). The IgG antibody levels to PPV23 were given as arbitrary units (U/mL), according to an in-house standard. All sera from a given patient were run on the same plate.

Serum antibody titers to influenza virus A/H1N1pdm09 were detected by influenza hemagglutination inhibition (HI) assay. This antigen was included in all the seasonal influenza vaccines given in this study. Sera were tested in serial two-fold dilutions starting at 1:10 with turkey red blood cells as indicator cells [38]. The HI titer is defined as the reciprocal value of the highest serum dilution that produces complete inhibition in the assay. An antibody titer ≥ 40 was considered protective [39].

### Statistical analysis

All statistical methods used were non-parametric. Differences in proportions were evaluated with chi-square tests. The effects of etoricoxib treatment were evaluated by comparing changes in variables (Δ) at four months of etoricoxib treatment between treatment arms within the ART-naïve and ART-treated groups separately. To increase statistical power, and as the wash-out period of etoricoxib effects on immunological parameters was considered to be in the order of days (t_1/2_ approx. 22 hours), the arms receiving two weeks of etoricoxib and no study drug were combined in analysis of immunological effects at four months. In analysis of vaccine responses at four weeks post-vaccination, the three treatment arms were analyzed separately, to evaluate the effect of short-term etoricoxib treatment as a vaccine adjuvant, compared to continuous etoricoxib therapy. COX-2i-related changes in immunological variables in all treatment arms are given in S1 Table. P-values <0.05 were considered statistically significant. No correction for multiple comparisons was made, to limit type II statistical errors in this exploratory trial.

## Results

### Patients included

A total of 28 ART naïve patients and 28 patients suppressed on stable ART were included in the study (clinical data in Table 1). After baseline, six patients in the ART naïve group interrupted the study, four due to an indication for ART initiation according to guidelines at the time and two due to non-serious adverse events (exacerbation of a pre-existing rash and oral blisters, respectively). One patient in the ART-treated group interrupted the study due to a deep vein thrombosis diagnosed a few days after commencing etoricoxib. Patient-reported compliance in taking etoricoxib was >95%.

**Table 1:** Clinical characteristics at baseline.

	ART naïve						ART-treated					
	ALL	Arm 1 (COX-2i 4 months)	Arm 2 (COX-2i 2 weeks)	Arm 3 (No COX-2i)	Arms 2/3	Arm 1 vs. Arm 2/3	ALL	Arm 1 (COX-2i 4 months)	Arm 2 (COX-2i 2 weeks)	Arm 3 (No COX-2i)	Arms 2/3	Arm 1 vs. Arm 2/3
N	28	13	7	8	15	p-value	28	13	8	7	15	p-value
**CD4 T cell count** (cells/mL)	**529 (477–696)**^**1**^	493 (450–545)	643 (562–819)	595 (465–1036)	643 (509–819)	***0*.*04***^***2***^	**364 (288–444)**	321 (228–379)	362 (309–415)	443 (344–523)	384 (340–458)	***0*.*07***
**CD4/CD8 ratio**	**0.46 (0.30–0.58)**	0.39 (0.30–0.47)	0.57 (0.40–0.64)	0.58 (0.35–0.70)	0.57 (0.40–0.64)	***0*.*03***	**0.48 (0.35–0.62)**	0.46 (0.30–0.48)	0.57 (0.43–0.63)	0.53 (0.35–0.99)	0.54 (0.39–0.65)	***0*.*10***
**Plasma HIV RNA** (copies/mL)	**24000 (10750–84000)**	35000 (14000–85000)	20000 (1700–83000)	24000 (12850–105500)	22000 (8700–83000)	*0*.*66*	**<50**	<50	<50	<50	<50	*1*.*0*
**Nadir CD4 T cell count** (cells/mL)	**496 (438–595)**	491 (441–545)	547 (452–590)	522 (393–628)	531 (425–622)	*0*.*56*	**169 (115–233)**	139 (64–226)	155 (118–210)	231 (180–249)	200 (136–234)	*0*.*39*
**Age (years)**	**41 (38–43)**	42 (39–43)	41 (34–49)	40 (36–41)	41 (34–42)	*0*.*25*	**44 (40–53)**	48 (41–51)	44 (39–49)	43 (42–57)	43 (39–54)	*0*. *91*
**Gender** (M/F)	**25/3**	12/1	7/0	6/2	13/2	*n*.*s*.^*3*^	**26/2**	12/1	7/1	7/0	14/1	*n*.*s*.
**Time since HIV diagnosis** (years)	**2.1 (1.0–5.3)**	1.9 (0.8–5.1)	3.1 (0.4–5.5)	2.2 (1.3–4.6)	2.4 (1.2–5.5)	*0*.*63*	**6.4 (3.4–10.7)**	5.7 (2.7–10.7)	3.9 (3.4–9.4)	8.2 (5.5–12.0)	6.8 (3.5–9.4)	*0*.*73*
**Time on ART** (years)	**-**	-	-	-	-		**4.3 (2.4–6.5)**	4.8 (2.6–5.6)	3.1 (1.7–5.4)	6.0 (2.5–7.9)	3.73 (2.3–7.0)	*0*.*50*

^1^Data given as median and lower/upper quartile

^2^ Mann-Whitney U test

^3^ Chi-square test

### Modified analysis due to baseline difference in CD4 count but no effect of COX-2i on clinical variables

**Fig 3:**
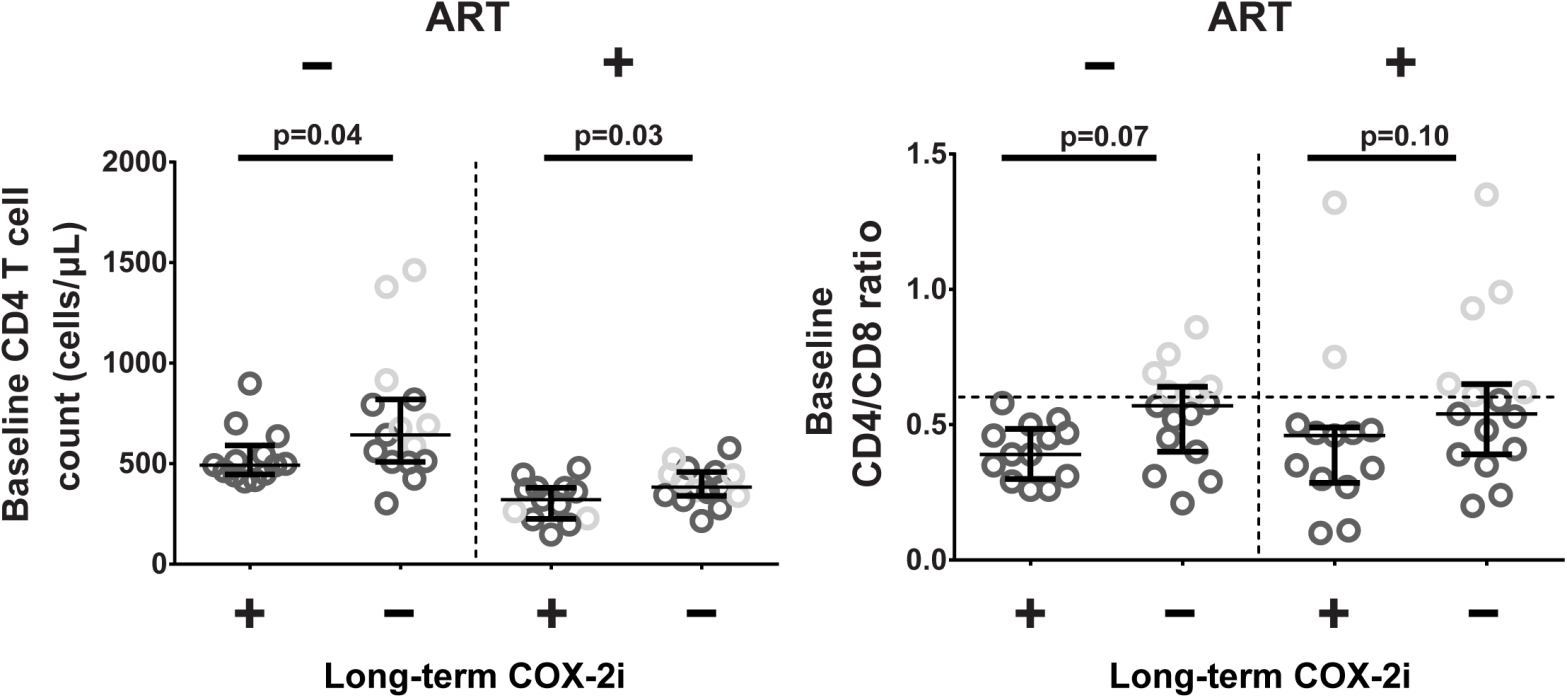
Baseline CD4^+^ T cell count and CD4/CD8 ratio. P-values derived from comparison of all included patients. For analysis of immunological effects of COX-2i after four months, arms 2 and 3 were combined (denoted Long-term COX-2i -), and patients with a CD4/CD8 ratio >0.6 (light grey color) were excluded. Median and lower/upper quartile indicated.

Comprehensive analysis of etoricoxib effects showed minimal differences between patients receiving long-term etoricoxib (arm 1) and controls (arm 2+3) within both the ART-naïve and ART-treated study groups. However, despite randomization, there were significant baseline differences in CD4 counts (p = 0.04) and CD4/CD8 ratio (p = 0.03) between treatment arms in the ART-naïve study group and similar evidence for a more advanced disease phenotype in ART-treated patients (Fig 3). While this baseline difference in key immunological markers was likely due to chance, we hypothesised that that it might significantly impact on study outcomes. Further analysis of etoricoxib effects after four months was therefore restricted to patients with a CD4/CD8 ratio < 0.6 (75^th^ percentile in the whole cohort), rendering all baseline clinical data baseline similar between the intervention arms, including CD4 counts and CD4/CD8 ratios (ART-naïve: median CD4 count 493 vs. 513, p = 0.39 and median CD4/CD8 ratio 0.39 vs. 0.45, p = 0.53, respectively. ART-treated: median CD4 count 361 vs. 358, p = 0.45 and median CD4/CD8 ratio 0.35 vs. 0.41, p = 0.31, respectively).

We nevertheless observed no effect of four months of etoricoxib treatment on CD4 counts or CD4/CD8 ratio, in ART-naïve or ART-treated patients. There was no effect of etoricoxib on HIV viremia in ART-naïve patients, or on viral load blips in ART-treated patients (data not shown).

### COX-2i reduces CD38 density in ART-naïve patients

As expected, the ART-naïve group had higher baseline expression of the activation marker CD38 on CD8^+^ T cells, both as frequency of positive cells and as CD38 density (molecules/cell, p<0.001, and tended to have higher expression of PD-1 on CD8^+^ T cells (% positive, p = 0.06) than the ART-treated group (Table 2).

**Table 2:** Baseline immunological parameters.

	ART-naïve			Arm 1 vs. Arm 2/3	ART-treated			Arm 1 vs. Arm 2/3	ART- vs. ART+
	ALL	Arm 1 (COX-2i 4 months)	Arms 2/3	p-value	ALL	Arm 1 (COX-2i 4 months)	Arms 2/3	p-value	p-value
**CD38 density on CD8**^**+**^ **T cells** (10^3^ molec./cell)	2.42 (2.03–2.74)^1^	2.63 (2.14–2.83)	2.34 (2.00–2.55)	*0*.*15*^2^	1.76 (1.45–1.92)	1.65 (1.45–1.79)	1.79 (1.43–1.98)	*0*.*42*	***<0*.*001***
**CD38 expression on CD8**^**+**^ **T cells** (%)	51.0 (40.1–64.3)	55.8 (42.1–65.3)	51.0 (38.0–60.4)	*0*.*66*	23.7 (19.8–32.3)	29.2 (20.3–32.6)	22.9 (16.7–31.5)	*0*.*42*	***<0*.*001***
**CD38 density on CD8**^**+**^**PD-1**^**+**^ **T cells** (10^3^ molec./cell)	3.24 (2.68–3.71)	3.36 (2.71–4.14)	2.97 (2.56–3.31)	*0*.*21*	2.28 (1.76–2.57)	2.28 (1.74–2.4)	2.31 (1.81–2.57)	*0*.*95*	***<0*.*001***
**CD38 density on CD8**^**+**^**HLA-DR**^**+**^ **T cells** (10^3^ molec./cell)	5.61 (4.70–7.30)	5.89 (5.15–7.49)	5.53 (4.49–6.59)	*0*.*45*	3.22 (2.54–4.03)	3.04 (2.31–4.13)	3.28 (2.80–3.92)	*0*.*48*	***<0*.*001***
**PD-1 expression on CD8**^**+**^ **T cells (%)**	25.4 (14.1–34.3)	26.9 (19.2–29.3)	22.5 (14–34.8)	*0*.*95*	17.4 (14.8–22.8)	16.3 (13–22.6)	20 (14.8–23.5)	*0*.*37*	***0*.*06***
**ETP** (%)	97.3 (85.2–109.3)	103.3 (93.4–112.9)	95.2 (84.3–101.9)	*0*.*12*	88.6 (80.35–97.1)	89.8 (78.2–97.4)	87.9 (83.7–93.0)	*0*.*91*	***0*.*013***
**Peak** (%)	104.3 (89.8–118.1)	107.9 (103.4–121.5)	95.6 (77.6–115.6)	*0*.*13*	83.1 (74.1–97.5)	85.2 (71.5–94.6)	82.7 (75.8–105.7)	*0*.*50*	***0*.*004***
**Plasma free TFPI** (ng/mL)	12.89 (9.36–15.75)	12.20 (9.52–14.37)	12.96 (9.21–16.10)	*0*.*42*	12.72 (10.69–14.72)	10.90 (10.54–14.30)	12.87 (11.57–15.49)	*0*.*47*	*0*.*83*
**Plasma free Protein S** (%)	79.56 (71.90–92.26)	90.01 (70.50–92.46)	78.76 (73.60–89.22)	*0*.*51*	91.18 (79.64–95.75)	91.99 (83.70–94.67)	90.36 (76.59–96.74)	*0*.*90*	***0*.*08***
**Plasma D-dimer** (ng/mL)	215.6 (135.2–257.1)	215.5 (171.8–252.6)	215.7 (116.3–261.6)	*0*.*77*	224.9 (152.3–262.7)	185.5 (157.1–253.5)	233.3 (105.4–286.7)	*0*.*63*	*0*.*68*
**Plasma sCD25** (pg/mL)	698 (340–1046)	734 (584–1046)	629 (281–1231)	*0*.*26*	303 (239–422)	311 (249–370)	279 (227–462)	*0*.*80*	***<0*.*001***
**Plasma sCD163** (μg /mL)	1.61 (1.39–1.86)	1.68 (1.52–1.79)	1.58 (1.32–1.87)	*0*.*48*	1.22 (1.03–1.57)	1.25 (1.06–1.61)	1.21 (0.92–1.49)	*0*.*35*	***<0*.*001***
**Plasma sCD14** (μg/mL)	20.2 (18.2–24.6)	20.6 (18.8–23.8)	20.1 (17.1–25.4)	*0*.*80*	22.4 (19.0–24.4)	21.6 (19.3–23.6)	22.8 (18.8–26.0)	*0*.*22*	*0*.*58*
**Plasma IL-6** (pg/mL)	0.78 (0.46–1.61)	0.83 (0.48–1.451)	0.72 (0.27–2.33)	*0*.*75*	0.75 (0.52–1.16)	0.78 (0.35–0.92)	0.68 (0.54–1.40)	*0*.*93*	*0*.*57*
**Plasma IP10** (pg/mL)	129 (106–234)	177 (116–267)	120 (69.3–212)	*0*.*19*	63.5 (42.3–108)	63.9 (33.8–88.2)	63.1 (44.1–151)	*0*.*42*	***<0*.*001***
**Plasma CRP** (μg/mL)	10.1 (6.8–34.1)	9.9 (8.4–18.8)	10.2 (4.8–50.1)	*0*.*98*	13.1 (3.5–27.7)	7.0 (2.6–17.2)	13.6 (7.6–42.5)	***0*.*10***	*0*.*89*
**Plasma kynurenine** (μmol/L)	1.98 (1.66–2.53)	2.52 (1.85–2.56)	1.78 (1.62–2.08)	***0*.*01***	1.665 (1.47–1.93)	1.70 (1.37–1.96)	1.66 (1.48–1.90)	*0*.*71*	***0*.*003***
**Plasma tryptophan** (μmol/L)	61.2 (52.0–68.7)	61.6 (53.2–68.9)	60.7 (50.2–68.4)	*0*.*77*	59.4 (52.85–68.3)	59.8 (53.7–76.2)	56.9 (51.8–63.7)	*0*.*31*	*0*.*96*
**Plasma K/T ratio**	0.032 (0.027–0.040)	0.036 (0.033–0.042)	0.030 (0.026–0.035)	***0*.*03***	0.026 (0.023–0.032)	0.025 (0.022–0.026)	0.027 (0.025–0.038)	***0*.*05***	***0*.*004***
**Total Gag-specific CD8**^**+**^ **T cell response** (%)	2.21 (0.80–4.89)	2.10 (1.66–2.79)	2.37 (0.60–7.05)	*0*.*84*	0.82 (0.57–2.49)	0.88 (0.60–3.24)	0.74 (0.27–1.52)	*0*.*50*	***0*.*046***

^1 ^Data given as median and lower/upper quartile

^2^ Mann-Whitney U test

**Fig 4:**
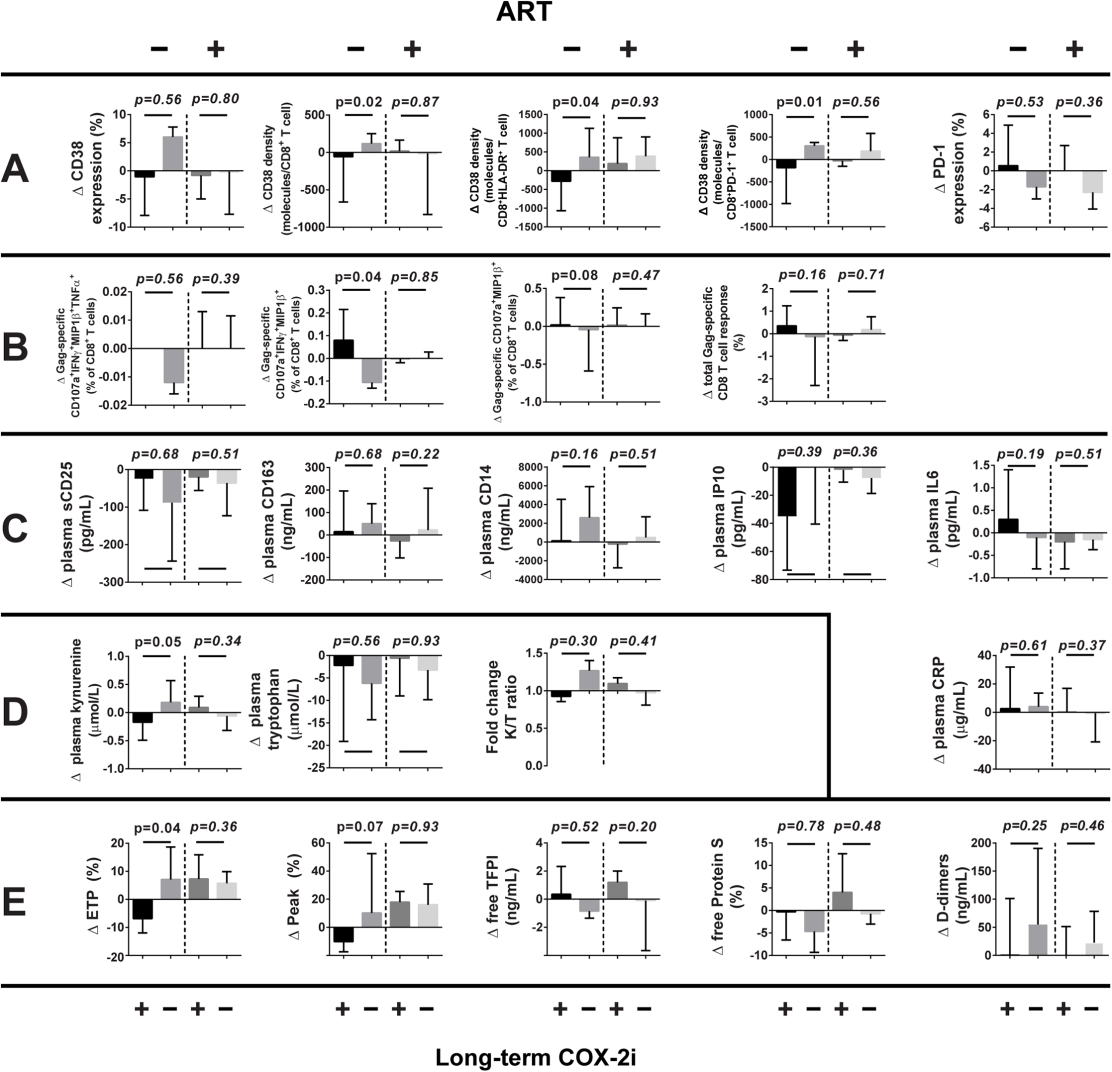
Change (Δ) in immunological variables from baseline to four months. ART use and long-term COX-2i treatment indicated. Data given as median and lower/upper quartile. A: T cell activation, B: Gag-induced CD8 T cell responses, C: Plasma inflammatory markers, D: Tryptophan metabolism, E: Coagulation.

In ART-naïve patients, long-term etoricoxib treatment reduced the density of activation marker CD38 in CD8^+^, CD8^+^HLADR^+^, and CD8^+^PD-1^+^ subsets (p = 0.015, p = 0.04 and p = 0.008, respectively, see Fig 4A). No effect on PD-1 expression on CD8^+^ T cells was observed (Fig 4A), nor on the relative frequency of naïve and memory CD8^+^ T cell subsets (data not shown). In contrast, etoricoxib did not significantly affect the expression of activation, exhaustion or differentiation markers on CD8^+^ T cells in ART-treated patients (Fig 4A).

Frequencies of both total (CD4^+^CD25^hi^CD127^lo^FoxP3^+^) and activated (CD4^+^CD25^hi^CD45RA^-^FoxP3^+^) Tregs were lower in the ART-naïve group than the ART-treated group at baseline (2.8 vs. 4.3%, p = 0.03 and 2.1 vs. 3.3%, p = 0.007, respectively), but there was no effect of etoricoxib on the frequency of Tregs in either study group (data not shown).

### COX-2i enhances HIV Gag-specific T cell responses

In terms of HIV-specific immunity, the baseline total CD8^+^ T cell response to Gag stimulation was higher in the ART-naive group (p = 0.046, Table 2). When the response was broken down into polyfunctional subsets and the prevalent subsets were analyzed separately, CD107a^+^MIP-1β^+^IFN-γ^+^ and CD107a^+^MIP-1β^+^ responses were significantly higher in the ART-naïve group compared with the ART-treated group (median 0.098% vs. 0.037%, p = 0.001 and median 0.066% vs. 0.038%, p = 0.04, respectively).

**Fig 5:**
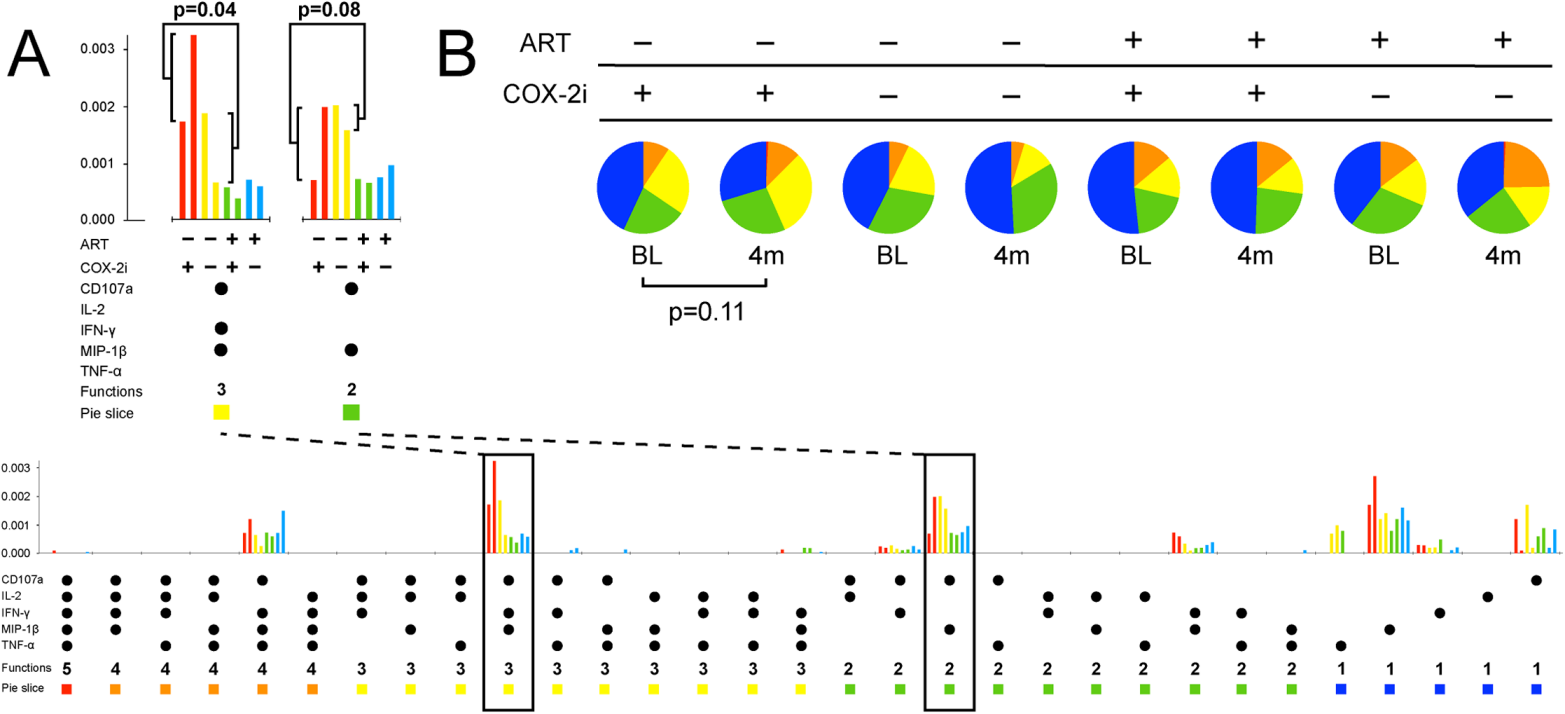
Polyfunctional responses of CD8^+^ T cells after stimulation with an HIV Gag peptide pool. A: Two prevalent polyfunctional subsets (CD107a^+^MIP1β^+^IFN-γ^+^ and CD107a^+^MIP1β^+^) increased after four months of etoricoxib in ART-naïve patients. ART and COX-2i use indicated, coloured bar pairs represent response at baseline and four months, respectively. B: The CD8^+^ T cell response tended to be more polyfunctional after four months of etoricoxib in ART-naïve patients (2 leftmost pies). P-values derived from unpaired permutation tests.

In ART-naïve patients, etoricoxib significantly increased the frequency of the HIV Gag-induced CD8^+^CD107a^+^MIP1β ^+^IFN-γ^+^ T cell subset (p = 0.04) and possibly increased the CD8^+^CD107a^+^MIP1β ^+^ subset (p = 0.08, Figs 4B and 5). Furthermore, the total Gag-induced T cell response as the proportion of CD8^+^ T cells appeared greater in ART-naïve patients treated with etoricoxib, but this effect was not statistically significant (p = 0.16, Fig 4B). No effect on HIV-specific T cell immunity was seen in the ART-treated study group.

### COX-2i does not modulate soluble markers of inflammation

Of the plasma soluble markers of inflammation assayed, the ART-naïve group had higher baseline levels of sCD25, IP10 and CD163 (all p<0.001), but not of CD14, IL-6 and CRP, than the ART-treated group (Table 2). There was, however, no effect of etoricoxib on any these markers in either patient group (Fig 4C).

### Effect of COX-2i on tryptophan metabolism

At baseline, the ART-naïve group had higher plasma levels of tryptophan catabolite kynurenine and a higher kynurenine/tryptophan (K/T) ratio than the ART-treated group (p = 0.003 and p = 0.004, respectively, Table 2). Etoricoxib significantly reduced plasma levels of kynurenine in ART-naive patients (p = 0.049, Fig 4D), but there was no significant effect on IDO activity, as defined by the K/T ratio. No effect on tryptophan metabolism was observed in ART-treated patients.

### COX-2i reduces *ex vivo* thrombin generation in ART-naïve patients

When plasma thrombin generation was assessed, ART-naïve patients had lower Endogenous Thrombin Potential (ETP) and Peak than ART-treated patients (p = 0.013 and p = 0.004, respectively, Table 2). There was some evidence for higher free Protein S in plasma in ART-naïve patients (p = 0.08), but no difference in plasma D-dimer or free TFPI.

In ART-naïve patients, etoricoxib treatment reduced ETP and there was a trend of reduced Peak (p = 0.04 and p = 0.08, respectively, Fig 4E), but there was no effect of etoricoxib on plasma levels of free TFPI, free Protein S or D-dimer. No effect of etoricoxib on any of the assayed coagulation parameters was observed in ART-treated patients.

### Effect of COX-2i on vaccine antibody responses

In the ART-naïve and ART-treated groups, respectively, 25 and 28 patients received a tetanus toxoid vaccine, 22 and 23 received a seasonal influenza vaccine, and 25 and 26 patients received a 13-valent conjugated pneumococcal vaccine.

**Fig 6:**
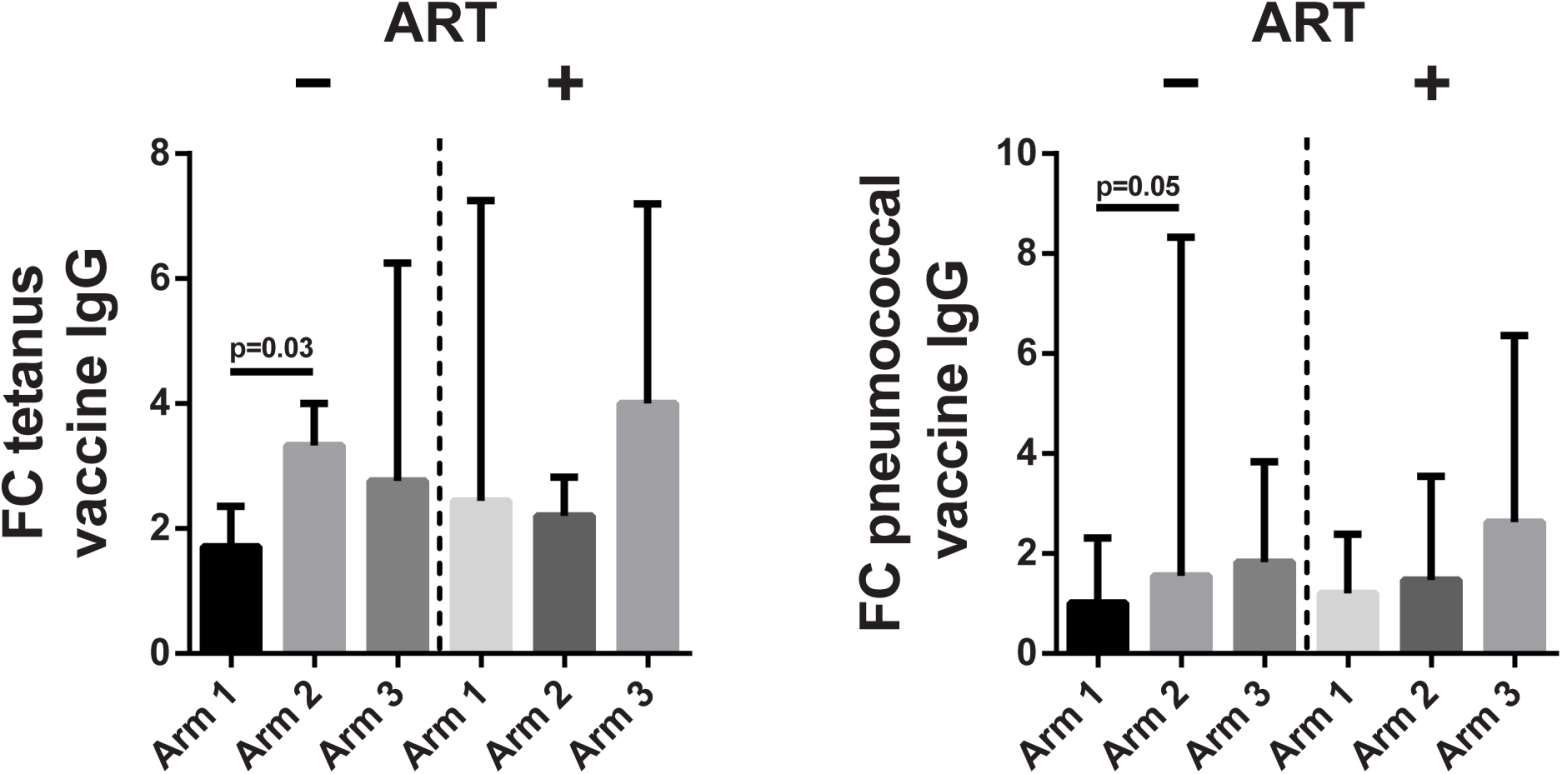
Vaccine IgG antibody responses. Fold change (FC) of tetanus toxoid and conjugated pneumococcal vaccine IgG levels in serum from baseline to 4 weeks post-vaccination, according to ART use and COX-2i treatment arm. All patients vaccinated in the study were included the analysis. Median and upper quartile indicated.

All patients but one had protective tetanus toxoid IgG levels of >0.1 IU/mL at baseline, indicating previous vaccination. Four weeks after vaccination, IgG levels increased by a factor of 2.4 (IQR 1.5–4.0, p<0.001) in both ART-naïve and ART-treated patients. ART-naïve patients receiving four months of etoricoxib had less of an antibody increase at week 4 post-vaccination than the arm receiving two weeks of etoricoxib (p = 0.03, Fig 6), with no difference between either treatment arm and the control arm. No significant effects of etoricoxib were found in the ART-treated study group.

Sixteen ART-naïve patients (57%) and 14 ART-treated patients (50%) had previously recorded vaccination with a 23-valent pneumococcal polysaccharide vaccine, but no patients had previously received the 13-valent conjugated pneumococcal vaccine. However, there was no significant difference in IgG levels at baseline or 4 weeks after administration of a 13-valent conjugated pneumococcal vaccine between previously vaccinated and unvaccinated patients (data not shown). Once again, ART-naïve patients receiving four months of etoricoxib had a smaller increase in IgG levels four weeks after vaccination than the corresponding two week etoricoxib arm (p = 0.05, Fig 6), while there was no difference between either etoricoxib arm and the control arm. No significant effect of etoricoxib treatment on pneumococcal vaccine IgG responses was found in the ART-treated study group.

At study baseline, 4 ART-naïve and 13 ART-treated patients (18% vs. 57%, respectively, p = 0.04) had protective influenza H1N1 IgG titers (>40), due to previous exposure to swine flu or vaccination with the same antigen. Of patients without pre-existing protective IgG levels, 11 ART-naïve and 7 ART-treated patients had protective IgG levels 4 weeks after vaccination (61% vs. 70%, p = 0.64), and no significant effect of etoricoxib treatment on influenza vaccine IgG levels was observed in either study group (data not shown).

## Discussion

The objective of this study was to explore the immunological effects of conventional doses of a COX-2 inhibitor, etoricoxib, in both untreated HIV-infected patients and in patients on ART with viral suppression but suboptimal CD4 reconstitution. In ART-naïve patients who received long-term etoricoxib, we observed a reduced density of activation marker CD38 on CD8^+^ T cells, enhanced HIV Gag-induced CD8^+^ T cell effector responses and reduced thrombin generation in plasma. However, in this patient group, no effects of etoricoxib on plasma markers of inflammation or tryptophan metabolism were observed. In this first study of a COX-2 inhibitor in ART-treated patients with suboptimal CD4 reconstitution, no significant effects of etoricoxib were observed.

Several markers of immune activation are predictive of clinical endpoints in HIV infection. In this and previous investigations of COX-2i, we quantified the density of CD38 molecules on CD8^+^ T cells and used this marker as a primary endpoint [31]. Although somewhat more laborious, this marker has been shown to be more predictive of disease progression than the more commonly used co-expression of CD38 and HLA-DR [6]. We have previously shown a reduction in CD38 density on CD8^+^ T cells in ART-naïve, HIV-infected patients after 12 weeks of high-dose celecoxib [27], but this was associated with a high incidence of rash. Our current data support that even a conventional, well-tolerated dose of COX-2i can reduce immune activation markers in ART-naïve patients. The 90 mg of etoricoxib qd administered in this study is a routine clinical dose used for pain relief in arthritic conditions, while patients in the celecoxib trial received 400mg bid, a dose only indicated in familial adenomatous polyposis.

In the current study, etoricoxib did not, however, reduce immune activation markers in aviremic patients on ART. In a previous study of rofecoxib and celecoxib in ART-treated patients, there was evidence of reduced CD38 expression on CD8 T cells only in the few patients with residual plasma viremia and higher baseline CD38 expression [32]. Thus, COX-2i may have a limited potential to further reduce immune activation in ART-treated patients with suppressed plasma viremia.

While T cell activation is a prerequisite for an effective adaptive immune response, the generalized hyperactivation seen in HIV infection is accompanied by compromised function, at least of HIV-specific T cell clones [15–17]. We hypothesized that COX-2i may improve HIV-specific T cell function by reducing PGE_2_ exposure of T cells, and did observe small increases in the relative frequency of prevalent polyfunctional Gag-induced CD8 subsets in ART-naïve patients who received etoricoxib. CD8 responses directed towards Gag epitopes have been repeatedly associated with viral control in untreated HIV infection [40–42] and polyfunctional CD8 responses are characteristic of patients with a non-progressor phenotype [43]. Thus, the apparent boosting of Gag responses we observe in this study may potentially prove beneficial for HIV-infected patients without access to ART. However, any clinical significance of these *in vitro* observations can only be ascertained in larger studies with longer observation periods.

HIV-associated inflammation is also reflected by elevated levels of plasma markers such as pro-inflammatory cytokine IL-6 and monocyte activation marker sCD14, which both predicted mortality in the SMART trial of intermittent vs. continuous ART [44, 45]. Comparing ART-naïve and ART-treated patients at baseline, we observed no difference in plasma levels of IL-6, sCD14 or CRP. This was unexpected in the case of IL-6, which has previously been found at higher levels in untreated patients [46], and CRP, which has in fact been found elevated in patients on suppressive ART compared to untreated patients [47]. The fact that the ART naïve patients in our study had relatively high CD4 counts while the ART-treated patients were selected based on suboptimal CD4 counts (median 529 vs. 364, respectively) may partly explain these observations. Our finding of comparable levels of sCD14 between patients off and on ART, however, is in line with other reports [48, 49]. Also consistent with these other studies was the elevated levels in ART-naïve patients of the other monocyte activation marker assayed, sCD163, as well as the more general markers of inflammation, IP10 and sCD25.

Treatment with etoricoxib, however, did not affect the plasma levels of these inflammatory markers, in ART-naïve or ART-treated patients. As CD14 is shed from monocytes on exposure to bacterial lipopolysaccharide (LPS), sCD14 is often used as a surrogate marker of microbial translocation from the gut [50]. The fact that etoricoxib did not affect plasma levels of sCD14 or any of the other inflammatory markers in this study may suggest limited effects of COX-2 inhibition on the gut barrier. This is in line with our previous study, where no change in plasma LPS was observed after celecoxib treatment of ART-naïve patients [31]. Interestingly, a short course of low-dose aspirin (an irreversible inhibitor of both COX-1 and COX-2) has been reported to reduce plasma sCD14, as well as CD38/HLA-DR expression on T cells and platelet reactivity in HIV-infected, ART-treated patients [51].

Another aspect of HIV-related immune dysregulation is the induction of the tryptophan-catabolizing enzyme IDO in innate immune cells by interferon-γ and Toll-like receptor stimulation. IDO upregulation has been implicated in the suppression of T cell responses as well as skewing the Th17/Treg balance towards increased frequencies of Tregs, which may contribute to gut barrier dysfunction and thus drive systemic inflammation in HIV infection [29]. IDO activity, as defined by the kynurenine/tryptophan ratio in plasma, is associated with progression of untreated HIV disease [29] and with poor CD4 reconstitution and mortality in ART-treated patients [52, 53]. In keeping with these studies, we observed a higher K/T ratio in ART-naïve than ART-treated patients. While there was a small reduction in plasma kynurenine in ART-naïve patients on etoricoxib, this effect was not significant when plasma tryptophan was taken into account. Furthermore, there was no effect of etoricoxib on IDO activity in ART-treated patients, nor any etoricoxib-related change in circulating Treg frequencies after etoricoxib treatment in either group.

HIV infection is associated with a state of hypercoagulability [54, 55], and elevated plasma levels of the fibrin degradation product D-dimer were strongly associated with both cardiovascular disease and all-cause mortality in the SMART trial [45, 56]. At baseline, we here found evidence of a pro-thrombotic phenotype in ART-naïve compared to ART-treated patients, which is in accordance with a number of previous studies, including SMART [57–59]. Somewhat surprisingly, and in contrast to previous studies, we did not measure lower plasma D-dimer in ART-treated patients. Once again, the large difference in baseline CD4 counts between ART-naïve and ART-treated patients in our study may have contributed to this.

COX-2i and most other non-steroidal anti-inflammatory drugs are associated with a small increase in the risk of cardiovascular events, particularly in patients with established cardiovascular disease [60]. However, we hypothesized that the anti-inflammatory effect of COX-2i could result in a net positive effect on cardiovascular risk in HIV-infected patients. In support of this notion, we did observe a reduction in thrombin generation in ART-naïve patients receiving etoricoxib. This may indicate a reduced risk of thromboembolic events, although the clinical significance of *in vitro* thrombin generation, particularly in hypercoagulable states, has not been firmly established [61].

A final hypothesis was that in the setting of HIV-associated immune hyperactivation, COX-2i may improve T cell help in stimulating B cells to produce vaccine antibodies. In keeping with this, we have previously observed improved IgG recall responses after tetanus toxoid vaccination in ART-naïve, HIV-infected patients receiving high-dose celecoxib [31]. However, in the current study long-term COX-2i therapy was associated with the poorest IgG responses to both tetanus toxoid and conjugated pneumococcal vaccines. While this discrepancy may be due to a lower dose of COX-2i in this study, a number of *in vitro* studies suggest that COX-2i have the potential to directly inhibit antibody production in B cells [62–64], which may trump T cell help in influencing the vaccine antibody response. An alternative explanation, given the improved Gag-induced CD8^+^ T cell responses we observed in ART-naïve etoricoxib-treated patients, is that COX-2i facilitate a Th1-polarization of the T cell response, reducing B cell help by T follicular helper cells. This could imply a potential enhancement of vaccine-specific CD8^+^ T cell responses with COX-2i treatment. Thus, our data suggest that COX-2i may modulate humoral and cellular vaccine responses differently in HIV-infected patients, which warrants further investigation in future studies.

This exploratory trial included a limited number of patients in several arms and the endpoint analysis unfortunately had to be performed after four as opposed to the per-protocol six months. Both these factors limit the study’s power to determine moderate effects of etoricoxib. The baseline differences in CD4 count and CD4/CD8 ratio between the treatment arms were unfortunate, and could potentially have been avoided by incorporating stratification on baseline CD4 count into the randomization procedure [65]. While the purpose of our modified analysis was to make the treatment arms more immunologically comparable, excluding patients in this manner may potentially negate some of the benefits of randomization. Furthermore, given the analysis of multiple primary and secondary endpoints, marginally significant p-values must be interpreted with caution.

Considering our hypothesis that PGE_2_ from activated innate immune cells plays a central role in COX-2-related immunomodulation, the study would have been strengthened by a characterisation of circulating monocytes and dendritic cells, particularly with regard to activation status, COX-2 expression and functional measures such as *in vitro* eicosanoid secretion in response to TLR agonists. Quantifying circulating levels of PGE_2_ and metabolites may also be relevant in future studies, although interpretation may be difficult due to short plasma half-lives [66] and whether plasma levels reflect biological effects in lymphoid tissues.

In conclusion, our results suggest that a conventional dose of a COX-2 inhibitor is safe in HIV-infected patients both on and off ART, and has potentially beneficial effects in untreated patients, on both generalized hyperactivation and HIV-specific function of CD8^+^ T cells, as well as a possible antithrombotic effect. We did not, however, observe comparable effects in patients with suboptimal immune reconstitution despite viral suppression on ART. While ART is now recommended for all HIV-infected patients, and global ART coverage is steadily improving, COX-2i or non-selective COX inhibitors could play a role in limiting immunological pathology in selected patients not accessing ART. However, the large clinical trials needed to establish any clinically relevant benefit of COX-2i treatment in this setting may not be feasible.

## Supporting information

S1 Fig Flow cytometry gating strategy. (TIF)

S1 File CONSORT Checklist. (DOC)

S2 File Study Protocol. (PDF)

S1 Table Changes in immunological variables in all treatment arms. (DOCX)

S2 Table Raw study data. (XLSX)

## References

[1] Paiardini M, Muller-Trutwin M. HIV-associated chronic immune activation. Immunol Rev. 2013;254: 78–101. doi: 10.1111/imr.12079 23772616 10.1111/imr.12079 PMC3729961

[2] Giorgi JV, Hultin LE, McKeating JA, Johnson TD, Owens B, Jacobson LP, et al Shorter survival in advanced human immunodeficiency virus type 1 infection is more closely associated with T lymphocyte activation than with plasma virus burden or virus chemokine coreceptor usage. J Infect Dis. 1999;179: 859–70. doi: 10.1086/314660 10068581 10.1086/314660

[3] Deeks SG, Kitchen CM, Liu L, Guo H, Gascon R, Narvaez AB, et al Immune activation set point during early HIV infection predicts subsequent CD4+ T-cell changes independent of viral load. Blood. 2004;104: 942–7. doi: 10.1182/blood-2003-09-3333 15117761 10.1182/blood-2003-09-3333

[4] Kuller LH, Tracy R, Belloso W, Wit SD, Drummond F, Lane HC, et al Inflammatory and Coagulation Biomarkers and Mortality in Patients with HIV Infection. PLoS Med. 2008;5:e203 doi: 10.1371/journal.pmed.0050203 18942885 10.1371/journal.pmed.0050203 PMC2570418

[5] Rodger AJ, Fox Z, Lundgren JD, Kuller LH, Boesecke C, Gey D, et al Activation and coagulation biomarkers are independent predictors of the development of opportunistic disease in patients with HIV infection. J Infect Dis. 2009;200: 973–83. doi: 10.1086/605447 19678756 10.1086/605447 PMC2892757

[6] Liu Z, Cumberland WG, Hultin LE, Prince HE, Detels R, Giorgi JV. Elevated CD38 antigen expression on CD8+ T cells is a stronger marker for the risk of chronic HIV disease progression to AIDS and death in the Multicenter AIDS Cohort Study than CD4+ cell count, soluble immune activation markers, or combinations of HLA-DR and CD38 expression. J Acquir Immune Defic Syndr Hum Retrovirol. 1997;16: 83–92. 9358102 10.1097/00042560-199710010-00003

[7] Hunt PW, Martin JN, Sinclair E, Bredt B, Hagos E, Lampiris H, et al T cell activation is associated with lower CD4+ T cell gains in human immunodeficiency virus-infected patients with sustained viral suppression during antiretroviral therapy. J Infect Dis. 2003;187: 1534–43. doi: 10.1086/374786 12721933 10.1086/374786

[8] Dyrhol-Riise AM, Voltersvik P, Olofsson J, Asjo B. Activation of CD8 T cells normalizes and correlates with the level of infectious provirus in tonsils during highly active antiretroviral therapy in early HIV-1 infection. AIDS. 1999;13: 2365–76. 10597778 10.1097/00002030-199912030-00008

[9] Hunt PW, Brenchley J, Sinclair E, McCune JM, Roland M, Page-Shafer K, et al Relationship between T cell activation and CD4+ T cell count in HIV-seropositive individuals with undetectable plasma HIV RNA levels in the absence of therapy. J Infect Dis. 2008;197: 126–33. doi: 10.1086/524143 18171295 10.1086/524143 PMC3466592

[10] Nakanjako D, Ssewanyana I, Mayanja-Kizza H, Kiragga A, Colebunders R, Manabe YC, et al High T-cell immune activation and immune exhaustion among individuals with suboptimal CD4 recovery after 4 years of antiretroviral therapy in an African cohort. BMC Infect Dis. 2011;11: 43 doi: 10.1186/1471-2334-11-43 21299909 10.1186/1471-2334-11-43 PMC3065409

[11] Freiberg MS, Chang CC, Kuller LH, Skanderson M, Lowy E, Kraemer KL, et al HIV infection and the risk of acute myocardial infarction. JAMA Intern Med. 2013;173: 614–22. doi: 10.1001/jamainternmed.2013.3728 23459863 10.1001/jamainternmed.2013.3728 PMC4766798

[12] Fultz SL, McGinnis KA, Skanderson M, Ragni MV, Justice AC. Association of venous thromboembolism with human immunodeficiency virus and mortality in veterans. Am J Med. 2004;116: 420–3. doi: 10.1016/j.amjmed.2003.10.011 15006592 10.1016/j.amjmed.2003.10.011

[13] Betts MR, Krowka JF, Kepler TB, Davidian M, Christopherson C, Kwok S, et al Human immunodeficiency virus type 1-specific cytotoxic T lymphocyte activity is inversely correlated with HIV type 1 viral load in HIV type 1-infected long-term survivors. AIDS Res Hum Retroviruses. 1999;15: 1219–28. doi: 10.1089/088922299310313 10480635 10.1089/088922299310313

[14] Edwards BH, Bansal A, Sabbaj S, Bakari J, Mulligan MJ, Goepfert PA. Magnitude of functional CD8+ T-cell responses to the gag protein of human immunodeficiency virus type 1 correlates inversely with viral load in plasma. J Virol. 2002;76: 2298–305. 11836408 10.1128/jvi.76.5.2298-2305.2002 PMC135950

[15] Shankar P, Russo M, Harnisch B, Patterson M, Skolnik P, Lieberman J. Impaired function of circulating HIV-specific CD8(+) T cells in chronic human immunodeficiency virus infection. Blood. 2000;96: 3094–101. 11049989

[16] Migueles SA, Weeks KA, Nou E, Berkley AM, Rood JE, Osborne CM, et al Defective human immunodeficiency virus-specific CD8+ T-cell polyfunctionality, proliferation, and cytotoxicity are not restored by antiretroviral therapy. J Virol. 2009;83: 11876–89. doi: 10.1128/JVI.01153-09 19726501 10.1128/JVI.01153-09 PMC2772718

[17] Lozano JM, De la Rosa O, Garcia-Jurado G, Luque J, Solana R, Kindelan JM, et al Impaired response of HIV type 1-specific CD8(+) cells from antiretroviral-treated patients. AIDS Res Hum Retroviruses. 2007;23: 1279–82. doi: 10.1089/aid.2007.0082 17961116 10.1089/aid.2007.0082

[18] International AIDS Society Scientific Working Group on HIV Cure; Deeks SG, Autran B, Berkhout B, Benkirane M, Cairns S, et al Towards an HIV cure: a global scientific strategy. Nat Rev Immunol. 2012;12: 607–14. doi: 10.1038/nri3262 22814509 10.1038/nri3262 PMC3595991

[19] Deng K, Pertea M, Rongvaux A, Wang L, Durand CM, Ghiaur G, et al Broad CTL response is required to clear latent HIV-1 due to dominance of escape mutations. Nature. 2015;517: 381–5. doi: 10.1038/nature14053 25561180 10.1038/nature14053 PMC4406054

[20] Shan L, Deng K, Shroff NS, Durand CM, Rabi SA, Yang HC, et al Stimulation of HIV-1-specific cytolytic T lymphocytes facilitates elimination of latent viral reservoir after virus reactivation. Immunity. 2012;36: 491–501. doi: 10.1016/j.immuni.2012.01.014 22406268 10.1016/j.immuni.2012.01.014 PMC3501645

[21] Brenchley JM, Price DA, Schacker TW, Asher TE, Silvestri G, Rao S, et al Microbial translocation is a cause of systemic immune activation in chronic HIV infection. Nat Med. 2006;12:1365–71. doi: 10.1038/nm1511 17115046 10.1038/nm1511

[22] Hinz B, Brune K, Pahl A. Cyclooxygenase-2 expression in lipopolysaccharide-stimulated human monocytes is modulated by cyclic AMP, prostaglandin E(2), and nonsteroidal anti-inflammatory drugs. Biochemic Biophys Res Commun. 2000;278:790–6. 10.1006/bbrc.2000.3885 11095985

[23] Lee IY, Cho W, Kim J, Park CS, Choe J. Human follicular dendritic cells interact with T cells via expression and regulation of cyclooxygenases and prostaglandin E and I synthases. J Immunol. 2008;180:1390–7. 18209033 10.4049/jimmunol.180.3.1390

[24] Endo Y, Blinova K, Romantseva T, Golding H, Zaitseva M. Differences in PGE2 Production between Primary Human Monocytes and Differentiated Macrophages: Role of IL-1β and TRIF/IRF3. PLoS One. 2014;9:e98517 doi: 10.1371/journal.pone.0098517 24870145 10.1371/journal.pone.0098517 PMC4037220

[25] Fogel-Petrovic M, Long JA, Knight DA, Thompson PJ, Upham JW. Activated human dendritic cells express inducible cyclo-oxygenase and synthesize prostaglandin E2 but not prostaglandin D2. Immunol Cell Biol. 2004;82:47–54. doi: 10.1111/j.1440-1711.2004.01213.x 14984594 10.1111/j.1440-1711.2004.01213.x

[26] Aandahl EM, Aukrust P, Muller F, Hansson V, Tasken K, Froland SS. Additive effects of IL-2 and protein kinase A type I antagonist on function of T cells from HIV-infected patients on HAART. AIDS. 1999;13: F109–14. 10597771 10.1097/00002030-199912030-00001

[27] Brudvik KW, Taskén K. Modulation of T cell immune functions by the prostaglandin E(2)–cAMP pathway in chronic inflammatory states. Br J Pharmacol. 2012;166: 411–9. doi: 10.1111/j.1476-5381.2011.01800.x 22141738 10.1111/j.1476-5381.2011.01800.x PMC3417476

[28] Mahic M, Yaqub S, Johansson CC, Tasken K, Aandahl EM. FOXP3+CD4+CD25+ adaptive regulatory T cells express cyclooxygenase-2 and suppress effector T cells by a prostaglandin E2-dependent mechanism. J Immunol. 2006;177: 246–54. 16785520 10.4049/jimmunol.177.1.246

[29] Favre D, Mold J, Hunt PW, Kanwar B, Loke Pn, Seu L, et al Tryptophan Catabolism by Indoleamine 2,3-Dioxygenase 1 Alters the Balance of TH17 to Regulatory T Cells in HIV Disease. Sci Transl Med. 2010;2: 32ra6. 10.1126/scitranslmed.3000632 PMC3034445 20484731

[30] Munn DH, Mellor AL. Indoleamine 2,3 dioxygenase and metabolic control of immune responses. Trends Immunol. 2013;34: 137–43. doi: 10.1016/j.it.2012.10.001 23103127 10.1016/j.it.2012.10.001 PMC3594632

[31] Pettersen FO, Torheim EA, Dahm AE, Aaberge IS, Lind A, Holm M, et al An exploratory trial of cyclooxygenase type 2 inhibitor in HIV-1 infection: downregulated immune activation and improved T cell-dependent vaccine responses. J Virol. 2011;85: 6557–66. doi: 10.1128/JVI.00073-11 21490090 10.1128/JVI.00073-11 PMC3126508

[32] Kvale D, Ormaasen V, Kran AM, Johansson CC, Aukrust P, Aandahl EM, et al Immune modulatory effects of cyclooxygenase type 2 inhibitors in HIV patients on combination antiretroviral treatment. AIDS. 2006;20: 813–20. doi: 10.1097/01.aids.0000218544.54586.f1 16549964 10.1097/01.aids.0000218544.54586.f1

[33] European AIDS Clinical Society. Guidelines v6.0. 2011. Available from: http://www.eacsociety.org/files/2011_eacsguidelines-v6.0-english_oct.pdf.

[34] Iyer SB, Hultin LE, Zawadzki JA, Davis KA, Giorgi JV. Quantitation of CD38 expression using QuantiBRITE beads. Cytometry. 1998;33: 206–12. 9773881 10.1002/(sici)1097-0320(19981001)33:2<206::aid-cyto15>3.0.co;2-y

[35] Roederer M, Nozzi JL, Nason MX. SPICE: Exploration and analysis of post-cytometric complex multivariate datasets. Cytometry A. 2011. 10.1002/cyto.a.21015 PMC3072288 21265010

[36] Hemker HC, Giesen P, Al Dieri R, Regnault V, de Smedt E, Wagenvoord R, et al Calibrated automated thrombin generation measurement in clotting plasma. Pathophysiol Haemost Thromb. 2003;33: 4–15. doi: 71636 12853707 10.1159/000071636

[37] Midttun O, Hustad S, Ueland PM. Quantitative profiling of biomarkers related to B-vitamin status, tryptophan metabolism and inflammation in human plasma by liquid chromatography/tandem mass spectrometry. Rapid Commun Mass Spectrom. 2009;23: 1371–9. doi: 10.1002/rcm.4013 19337982 10.1002/rcm.4013

[38] Waalen K, Kilander A, Dudman SG, Krogh GH, Aune T, Hungnes O. High prevalence of antibodies to the 2009 pandemic influenza A(H1N1) virus in the Norwegian population following a major epidemic and a large vaccination campaign in autumn 2009. Euro Surveill. 2010;15. 20738992

[39] Potter CW, Oxford JS. Determinants of immunity to influenza infection in man. Br Med Bull. 1979;35: 69–75. 367490 10.1093/oxfordjournals.bmb.a071545

[40] Geldmacher C, Currier JR, Herrmann E, Haule A, Kuta E, McCutchan F, et al CD8 T-cell recognition of multiple epitopes within specific Gag regions is associated with maintenance of a low steady-state viremia in human immunodeficiency virus type 1-seropositive patients. J Virol. 2007;81: 2440–8. doi: 10.1128/JVI.01847-06 17182686 10.1128/JVI.01847-06 PMC1865944

[41] Honeyborne I, Prendergast A, Pereyra F, Leslie A, Crawford H, Payne R, et al Control of human immunodeficiency virus type 1 is associated with HLA-B*13 and targeting of multiple gag-specific CD8+ T-cell epitopes. J Virol. 2007;81: 3667–72. doi: 10.1128/JVI.02689-06 17251285 10.1128/JVI.02689-06 PMC1866034

[42] Julg B, Williams KL, Reddy S, Bishop K, Qi Y, Carrington M, et al Enhanced anti-HIV functional activity associated with Gag-specific CD8 T-cell responses. J Virol. 2010;84: 5540–9. doi: 10.1128/JVI.02031-09 20335261 10.1128/JVI.02031-09 PMC2876607

[43] Betts MR, Nason MC, West SM, De Rosa SC, Migueles SA, Abraham J, et al HIV nonprogressors preferentially maintain highly functional HIV-specific CD8+ T cells. Blood. 2006;107: 4781–9. doi: 10.1182/blood-2005-12-4818 16467198 10.1182/blood-2005-12-4818 PMC1895811

[44] Sandler NG, Wand H, Roque A, Law M, Nason MC, Nixon DE, et al Plasma levels of soluble CD14 independently predict mortality in HIV infection. J Infect Dis. 2011;203:780–90. doi: 10.1093/infdis/jiq118 21252259 10.1093/infdis/jiq118 PMC3071127

[45] Kuller LH, Tracy R, Belloso W, De Wit S, Drummond F, Lane HC, et al Inflammatory and coagulation biomarkers and mortality in patients with HIV infection. PLoS Med. 2008;5: e203 doi: 10.1371/journal.pmed.0050203 18942885 10.1371/journal.pmed.0050203 PMC2570418

[46] Borges AH, O'Connor JL, Phillips AN, Ronsholt FF, Pett S, Vjecha MJ, et al Factors Associated With Plasma IL-6 Levels During HIV Infection. J Infect Dis. 2015;212: 585–95. doi: 10.1093/infdis/jiv123 25722296 10.1093/infdis/jiv123 PMC4598808

[47] Guimaraes MM, Greco DB, Figueiredo SM, Foscolo RB, Oliveira AR Jr., Machado LJ. High-sensitivity C-reactive protein levels in HIV-infected patients treated or not with antiretroviral drugs and their correlation with factors related to cardiovascular risk and HIV infection. Atherosclerosis. 2008;201: 434–9. doi: 10.1016/j.atherosclerosis.2008.02.003 18359028 10.1016/j.atherosclerosis.2008.02.003

[48] Mendez-Lagares G, Romero-Sanchez MC, Ruiz-Mateos E, Genebat M, Ferrando-Martinez S, Munoz-Fernandez MA, et al Long-term suppressive combined antiretroviral treatment does not normalize the serum level of soluble CD14. J Infect Dis. 2013;207: 1221–5. doi: 10.1093/infdis/jit025 23322858 10.1093/infdis/jit025

[49] Castley A, Berry C, French M, Fernandez S, Krueger R, Nolan D. Elevated plasma soluble CD14 and skewed CD16+ monocyte distribution persist despite normalisation of soluble CD163 and CXCL10 by effective HIV therapy: a changing paradigm for routine HIV laboratory monitoring? PLoS One. 2014;9: e115226 doi: 10.1371/journal.pone.0115226 25544986 10.1371/journal.pone.0115226 PMC4278884

[50] Sandler NG, Douek DC. Microbial translocation in HIV infection: causes, consequences and treatment opportunities. Nat Rev Microbiol. 2012;10: 655–66. doi: 10.1038/nrmicro2848 22886237 10.1038/nrmicro2848

[51] O'Brien M, Montenont E, Hu L, Nardi MA, Valdes V, Merolla M, et al Aspirin attenuates platelet activation and immune activation in HIV-1-infected subjects on antiretroviral therapy: a pilot study. J Acquir Immune Defic Syndr. 2013;63: 280–8. doi: 10.1097/QAI.0b013e31828a292c 23406976 10.1097/QAI.0b013e31828a292c PMC3756489

[52] Byakwaga H, Boum Y 2nd, Huang Y, Muzoora C, Kembabazi A, Weiser SD, et al The kynurenine pathway of tryptophan catabolism, CD4+ T-cell recovery, and mortality among HIV-infected Ugandans initiating antiretroviral therapy. J Infect Dis. 2014;210:383–91. doi: 10.1093/infdis/jiu115 24585899 10.1093/infdis/jiu115 PMC4148610

[53] Hunt PW, Sinclair E, Rodriguez B, Shive C, Clagett B, Funderburg N, et al Gut epithelial barrier dysfunction and innate immune activation predict mortality in treated HIV infection. J Infect Dis. 2014;210: 1228–38. doi: 10.1093/infdis/jiu238 24755434 10.1093/infdis/jiu238 PMC4192038

[54] Shen YM, Frenkel EP. Thrombosis and a hypercoagulable state in HIV-infected patients. Clin Appl Thromb Hemost. 2004;10: 277–80. 15247986 10.1177/107602960401000311

[55] Baker JV. Chronic HIV Disease and Activation of the Coagulation System. Thromb Res. 2013;132: 495–9. doi: 10.1016/j.thromres.2013.08.016 24034985 10.1016/j.thromres.2013.08.016 PMC4197841

[56] Duprez DA, Neuhaus J, Kuller LH, Tracy R, Belloso W, De Wit S, et al Inflammation, coagulation and cardiovascular disease in HIV-infected individuals. PLoS One. 2012;7: e44454 doi: 10.1371/journal.pone.0044454 22970224 10.1371/journal.pone.0044454 PMC3438173

[57] Baker JV, Brummel‐Ziedins K, Neuhaus J, Duprez D, Cummins N, Dalmau D, et al HIV Replication Alters the Composition of Extrinsic Pathway Coagulation Factors and Increases Thrombin Generation. J Am Heart Assoc. 2013;2: e000264 doi: 10.1161/JAHA.113.000264 23896681 10.1161/JAHA.113.000264 PMC3828789

[58] Jong E, Louw S, Meijers JC, de Kruif MD, ten Cate H, Buller HR, et al The hemostatic balance in HIV-infected patients with and without antiretroviral therapy: partial restoration with antiretroviral therapy. AIDS Patient Care STDS. 2009;23: 1001–7. doi: 10.1089/apc.2009.0173 19929230 10.1089/apc.2009.0173

[59] Musselwhite LW, Sheikh V, Norton TD, Rupert A, Porter BO, Penzak SR, et al Markers of endothelial dysfunction, coagulation and tissue fibrosis independently predict venous thromboembolism in HIV. AIDS. 2011;25: 787–95. doi: 10.1097/QAD.0b013e3283453fcb 21412059 10.1097/QAD.0b013e3283453fcb PMC4681576

[60] Bhala N, Emberson J, Merhi A, Abramson S, Arber N, Baron JA, et al Vascular and upper gastrointestinal effects of non-steroidal anti-inflammatory drugs: meta-analyses of individual participant data from randomised trials. Lancet. 2013;382: 769–79. doi: 10.1016/S0140-6736(13)60900-9 23726390 10.1016/S0140-6736(13)60900-9 PMC3778977

[61] van Veen JJ, Gatt A, Makris M. Thrombin generation testing in routine clinical practice: are we there yet? Br J Haematol. 2008;142: 889–903. doi: 10.1111/j.1365-2141.2008.07267.x 18564356 10.1111/j.1365-2141.2008.07267.x

[62] Chang C-L, Ma B, Pang X, Wu TC, Hung C-F. Treatment With Cyclooxygenase-2 Inhibitors Enables Repeated Administration of Vaccinia Virus for Control of Ovarian Cancer. Mol Ther. 2009;17:1365–72. doi: 10.1038/mt.2009.118 19471247 10.1038/mt.2009.118 PMC2835247

[63] Ryan EP, Malboeuf CM, Bernard M, Rose RC, Phipps RP. Cyclooxygenase-2 inhibition attenuates antibody responses against human papillomavirus-like particles. J Immunol. 2006;177: 7811–9. 17114452 10.4049/jimmunol.177.11.7811

[64] Ryan EP, Pollock SJ, Murant TI, Bernstein SH, Felgar RE, Phipps RP. Activated human B lymphocytes express cyclooxygenase-2 and cyclooxygenase inhibitors attenuate antibody production. J Immunol. 2005;174: 2619–26. 15728468 10.4049/jimmunol.174.5.2619

[65] Kernan WN, Viscoli CM, Makuch RW, Brass LM, Horwitz RI. Stratified Randomization for Clinical Trials. J Clin Epidemiol. 1999;52: 19–26. 9973070 10.1016/s0895-4356(98)00138-3

[66] Bygdeman M. Pharmacokinetics of prostaglandins. Best Pract Res Clin Obstet Gynaecol. 2003;17:707–16. 12972009 10.1016/s1521-6934(03)00043-9

