# Supplementary figures and images for: Immune activation and HIV-specific T cell responses are modulated by a cyclooxygenase-2 inhibitor in untreated HIV-infected individuals: An exploratory clinical trial
Source: PLoS One. 2017 May 2;12(5):e0176527. doi: 10.1371/journal.pone.0176527 (PMC5413033; doi:10.1371/journal.pone.0176527)

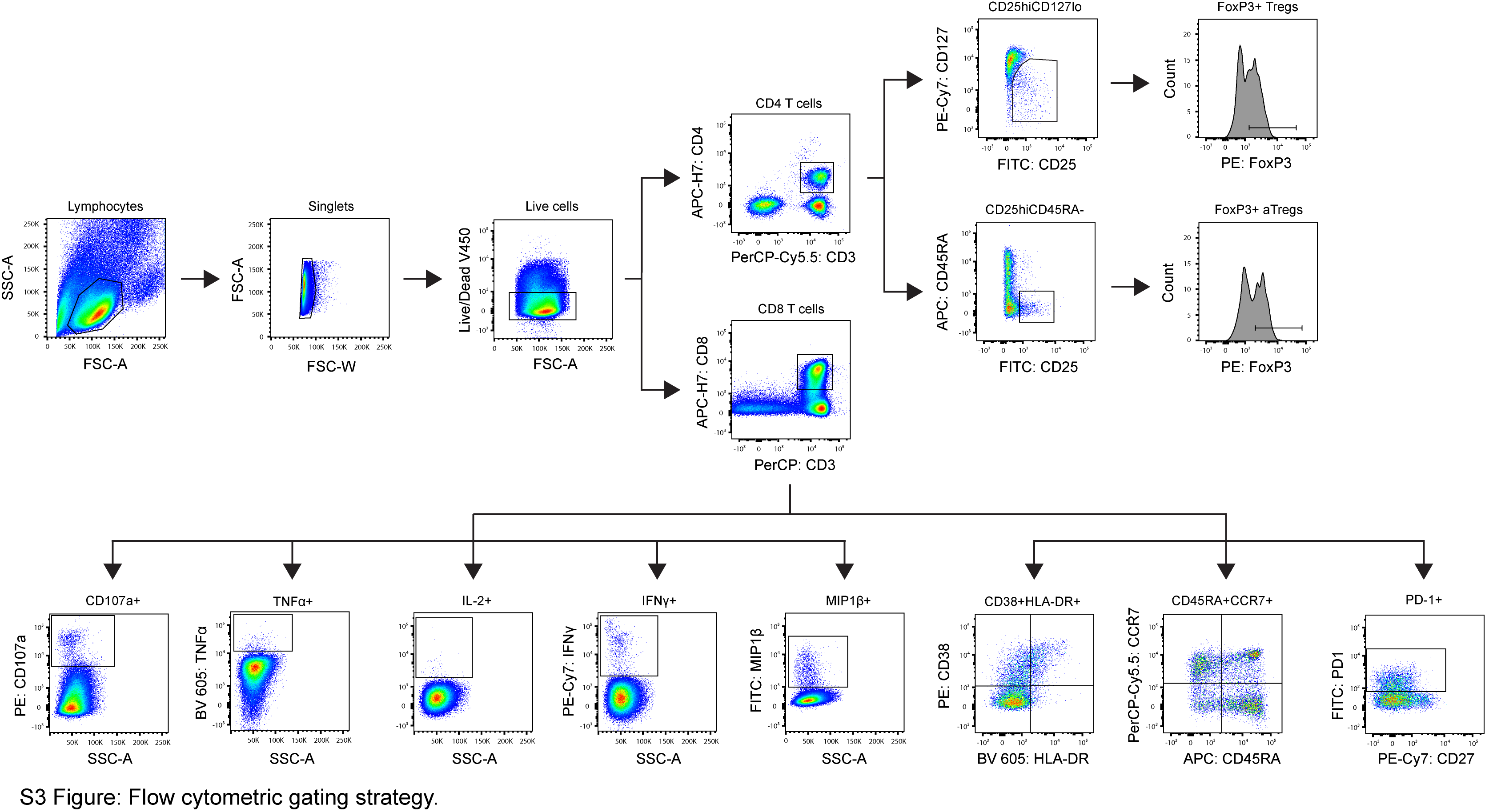

Supplement: S1 Fig — (TIF) [file pone.0176527.s001.tif]
