# Supplementary material for: Immune activation and HIV-specific T cell responses are modulated by a cyclooxygenase-2 inhibitor in untreated HIV-infected individuals: An exploratory clinical trial
Source: PLoS One. 2017 May 2;12(5):e0176527. doi: 10.1371/journal.pone.0176527 (PMC5413033; doi:10.1371/journal.pone.0176527)
Supplement: S2 File — (PDF) [file pone.0176527.s003.pdf]

Clinical Study Protocol

Optional immunomodulating therapy and improved vaccination responses by adjuvant administration of a cyclooxygenase type 2 inhibitor in HIV-infected patients

Protocol code:	OUSCOX2
EudraCT No:	2010-020382-25
Version:	5
Date:	21.05.2012

Principle Investigator:
Professor Dag Kvale, MD, PhD
Oslo University Hospital
Department of Infectious Diseases Ullevål
Oslo, Norway
dag.kvale@medisin.uio.no

Co-investigator:
Professor Kjetil Taskén, MD, PhD
The Biotechnology Centre
University of Oslo
Oslo, Norway
kjetil.tasken@biotek.uio.no

Confidentiality Statement
The information contained in this document is confidential and cannot be disclosed unless required by governmental regulation. Persons to whom any portion of the contents of this document is disclosed must be informed that the information is confidential and may not be further disclosed by them.

APPROVAL PAGE

Principal Investigator:

Prof. Dag Kvale, MD, PhD Date

Co-investigator:

Name: Date:
Prof. Kjetil Taskén, MD, PhD

PROTOCOL SYNOPSIS

**Name of Sponsor/Company:**<br><br>**Oslo University Hospital**
**Name of Products:**<br><br>Arcoxia (MSD (Norge) AS)
**Name of Active Ingredient:**<br><br>Etoricoxib
**Title of Study:**<br><br>Optional immunomodulating therapy and improved vaccination responses by adjuvant administration of a cyclooxygenase type 2 inhibitor in HIV-infected patients
**Protocol Number:**<br><br>OUSCOX2
**Investigators and Study Centre(s):** (Name, institution, city/state)<br><br>Professor Dag Kvale, MD, PhD, Oslo University Hospital, Oslo (PI)<br><br>To be determined by separate application: Karolinska Institute, Stockholm, Sweden, and Rigshospitalet, København, Denmark.
**Phase of Development:** Phase IIa
**Objectives:**<br><br>To explore the efficacy of etoricoxib on progression markers and vaccine responses in HIV-infected patients on and off antiretroviral treatment (ART).<br><br>To explore the safety of etoricoxib for 2 and 24 weeks, respectively in HIV-infected patients on effective ART with subnormal CD4 counts and off ART.
**Methodology:**<br><br>Open, controlled, randomized, multicentre
**Number of Subjects Planned:**<br><br>Approximately 20-30 patients without current indication for ART and 20-30 patients receiving ART will be included. A controlled clinical trial will be carried out within each stratum randomized (2:1:1) to 3 study arms; Arm #1 randomized to Arcoxia for 24 weeks (n=30), #2 to Arcoxia for 2 weeks (n=15), and Arm #3 as control (n=15). All groups will be vaccinated, at week 6 (Arm #1) or at week 1 (Arms #2 and #3, respectively).
**Diagnosis and Main Criteria for Inclusion:** Male and female patients between 18 and 65 years of age with a confirmed diagnosis of HIV infection < 8 years prestudy. For patients <u>without ART:</u> No HIV-related clinical manifestations including acute HIV infection; no current indication or use for antiretroviral treatment; CD4+ count > 350 x 10^6/l; HIV RNA > 2000 copies/ml. For patients on stable

effective ART > 12 months having viral suppression with HIV RNA < 50 copies/ml > 6 months and CD4+ count < 500 x 10^6/l.

Main exclusion criteria:

(i) Study-specific: Concomitant or sporadic use of NSAID, corticosteroids or other immune modulating therapies including interferon-alpha, cholesterol > 7 μM, under treatment for hypertension or antihypertensive treatment indicated at inclusion, cardiovascular events or stroke in parents, siblings or off-springs occurring < 55 years of age, elevated serum creatinine, diabetes type I or II; and **(ii) Exclusion criteria according to SPC:** Known hypersensitivity for etoricoxib, capsule substances or sulphonamides, active peptic ulcer or gastrointestinal haemorrhage, history of asthma, acute rhinitis, nasal polyps, angioneurotic oedema, urticaria or other allergic reactions after taking acetyl salicylic acid or NSAID including COX-2 inhibitors, pregnancy or insufficient birth control for females, breastfeeding, seriously deranged liver function, creatine clearance < 30 ml/min, inflammatory bowel disease, heart failure (NYHA II-IV), established ischaemic heart disease, peripheral arteriosclerosis and/or cerebrovascular disease.

Investigational Product, Dose and Mode of Administration:

Arcoxia (etoricoxib) tablets 90 mg, administered p.o. at 90 mg qd.

In the case of intolerable subjective adverse reactions, dose may be reduced to 60 mg qd or drug stopped.

Vaccines (seasonal influenza, Tetanus toxoid, Hepatitis A, conjugated pneumococcal vaccine and human papillomavirus (optional)) are all registered and safe to use in immune compromised hosts.

Study Duration*:*

Informed consent, screening, inclusion and baseline assessments at week -4 to week 0, followed by 24 weeks of study drug administration, post study drug follow-up control at week 36.

The total study duration is 36 weeks.

Criteria for Evaluation:

Efficacy: Changes in CD38 density (CD38 molecules per CD38+CD8+CD3+ T cells) and in humoral and cellular immune responses to study-specific vaccines (primary).

Changes in CD38 density (molecules/cell) in CD8+PD-1+ or other T cell subsets, CD4+ T cell counts, HIV RNA, immunoglobulin levels, β2-microglobulin, CRP, D-dimer. HIV-related clinical events, Indication for antiretroviral treatment (secondary).

Safety: Reductions of etoricoxib dose or stop of drug, adverse events including cardiovascular events, blood pressure, clinical chemistry, serum concentration of antiretroviral drugs.

Pharmacokinetics: None

Statistical Methods:

Non-parametrical methods to describe patient groups including matched pair statistics to determine primary endpoints within the four study arms

TABLE OF CONTENTS

Page No.

APPROVAL PAGE **2**
PROTOCOL SYNOPSIS **3**
TABLE OF CONTENTS **5**
LIST OF ABBREVIATIONS **7**
1 INTRODUCTION **8**
2 OBJECTIVES **10**
3 STUDY DESIGN **11**
3.1 Inclusion Criteria 12
3.2 Exclusion Criteria 12
3.2.1 Study-specific exclusion criteria 12
3.2.2 Exclusion criteria according to the Summary of Product Characteristics (SPC) on Arcoxia from Norwegian Medicines Agency 12
3.3 Withdrawal and Termination Criteria 13
4 TREATMENT **14**
4.1 Selection of Drugs and Doses 14
4.2 Treatments Administered 14
4.3 Investigational Product 14
4.3.1 Supply and Packaging 15
4.4 Method of Assigning Subjects to Treatment Groups 15
4.5 Randomization 15
4.6 Prior and Concomitant Therapy 16
4.7 Treatment Compliance 16
5 EFFICACY AND SAFETY VARIABLES **17**
5.1 Efficacy and Safety Measurements (Study Schematic) 17
5.2 Efficacy Assessments 17
5.2.1 Efficacy Variables 17
5.2.1.1 Primary Efficacy Endpoints 18
5.2.1.2 In vitro immune parameters 18
5.2.1.3 Immune parameters after in vivo vaccination 18
5.3 Safety Assessments 18
5.3.1 Medical History 19
5.3.2 Physical Examination 19
5.3.3 Vital Signs 19
5.3.4 Clinical Laboratory Evaluation 19
5.3.5 Adverse Events (AEs) 20
5.3.6 Serious Adverse Events (SAEs) 20
5.3.7 Adverse Event and Serious Adverse Event Reporting 20
6 DATA HANDLING AND QUALITY ASSURANCE **22**
6.1 Completing, Signing and Archiving Case Report Forms (CRFs) 22
6.2 Clinical Data Management 22
6.3 Archiving 22
7 STATISTICAL METHODS AND PLANNED ANALYSIS **23**
7.1 General Statistical Considerations 23
7.2 Subject Characteristics 23
7.2.1 Study Population Variables 23
7.3 Safety Analysis 23
7.3.1 Adverse Events 23
7.3.2 Vital Signs 23
7.3.3 Physical Examinations 23
7.3.4 Clinical Laboratory Measurements 23
7.4 Efficacy Analysis 24
7.5 Sample Size Determination 24
7.6 Significance Level 24
7.7 Procedures for Missing, Unused and Spurious Data 24

7.8 Rules for Excluding Subjects from Analysis 24
7.9 Procedures for Reporting Deviations from Original Statistical Plan 24
8 SPECIAL REQUIREMENTS AND PROCEDURES 25
8.1 Ethics Committee Review 25
8.2 Handling and storage of biological material 25
8.3 Changes to the Conduct of the Study or Protocol 25
8.4 Investigator’s Responsibilities 25
8.4.1 Overall Responsibilities 25
8.4.2 Subject Informed Consent 26
8.4.3 Direct Access to Source Data/Documents 26
8.4.4 Confidentiality Regarding Study Subjects 26
8.5 Study Monitoring 26
8.6 Audit and Inspection 26
9 INVESTIGATIVE AGREEMENT 27
9.1 Financial Disclosure 27
9.2 Confidentiality 27
9.3 Insurance 27
10 REFERENCES 28

LIST OF ABBREVIATIONS

AE	-	*adverse event*
AIDS	-	*acquired immunodeficiency syndrome*
ASA	-	*acetyl salicylic acid*
ASAT	-	*aspartate aminotransferase*
ALAT	-	*alanine aminotransferase*
ART	-	*antiretroviral treatment*
cAMP-		*cyclic adenosine mono phosphate*
CART -		*combination antiretroviral therapy*
CFR	-	*Code of Federal Regulations (USA)*
COX	-	*cyclooxygenase*
COX-2i -		*cyclooxygenase-2 inhibitors*
CK	-	*creatine kinase*
CRF	-	*case record form*
CRO	-	*contract research organization*
CRP	-	*C-reactive protein*
EC	-	*ethics committee*
GCP	-	*good clinical practice*
HAART -		*highly active antiretroviral treatment*
HIV	-	*human immunodeficiency virus*
ICH	-	*International Conference on Harmonization*
LDH	-	*lactate dehydrogenase*
LPS	-	*lipopolysaccharide*
NSAID-		*non-steroid anti-inflammatory drug*
PGE	-	*prostaglandin E*
PKA	-	*protein kinase A*
SEB	-	*staphylococcal enterotoxin B*
SOP	-	*standard operating procedure*
TNF	-	*tumor necrosis factor*

1 INTRODUCTION

Human immunodeficiency virus type 1 (HIV) causes a chronic infection leading to severe dysfunction of the immune system with a markedly increased incidence of opportunistic infections and certain forms of malignancies, ultimately leading to death. HIV-specific antiretroviral treatment (ART) has significantly improved the prognosis of HIV-infected patients. However, ART is not commonly available in many areas with high prevalence of HIV, and follow-up on ART requires a certain level of health care infrastructure.

Chronic immune activation accompanies chronic HIV infection and accelerates the development of immune deficiency. After primary HIV infection which is characterized by a substantial CD4+ T cell loss at mucosal sites and elsewhere (1), clinical progression in chronic HIV infection results from the inability of the immune system to control the viral replication (2, 3). Effective and specific polyfunctional T cell responses and T cell proliferative capacity are instrumental in controlling HIV (4). However, HIV mutagenesis and immune escape undermine the capacity of the immune system to maintain immune control. Numerous factors have also been shown to modulate the responsiveness of the immune system, including regulatory T cells (Treg) (5, 6), inhibitory soluble factors (7), as well as reversible dysfunction or exhaustion of T cells resulting from the expression of inhibitory surface receptors such as cytotoxic T lymphocyte antigen (CTLA)-4 and programmed death-1 (PD-1) (8, 9). These modulating mechanisms may all constitute both causes and consequences of the HIV-associated chronic immune activation (10). However, markers of chronic activation, such as CD38 on CD8+ T cells, appear to be equally or even better suited to predict clinical progression in HIV infection than HIV RNA levels and CD4+ T cell counts in untreated patients (8, 10-12). Notably, T cell activation is not normalized despite prolonged ART and is associated with poorer recovery of CD4+ T cells (35), and it was recently reported that activated T cells predict CD4+ recovery and mortality in patients receiving ART (36). **Targeting the HIV-related chronic immune activation may therefore be a therapeutic strategy *per se* both for untreated subjects and patients on effective ART with incomplete normalization of CD4 counts (immunological non-responders).**

The current trial was based on our observations that augmented levels of cyclic adenosine monophosphate (cAMP) contribute to the T cell dysfunction in HIV-infected patients (13-15). In T cells, cAMP triggers a protein kinase A (PKA) – Csk – Lck inhibitory pathway that inhibits the proximal T cell receptor (TCR) signaling events (13-14, 16-20). This mechanism may also be involved in the inhibitory function of Tregs (21). We have hypothesized that elevated levels of cAMP in T cells from HIV-infected individuals result from increased production of prostaglandin E_2 (PGE_2) following activation-induced expression of cyclooxygenase type 2 (COX-2) in lymphoid tissues. Although we have identified even COX-2 positive T cells in HIV-infected individuals (22), activated monocytes may be the major source of PGE_2 (23); high levels of COX-2 are produced *de novo* after a number of stimuli, particularly lipopolysaccharide (LPS) (24, 25). Circulating LPS is indeed increased in untreated chronic HIV infection due to enhanced translocation of microbial material and correlates to chronic immune activation and disease progression (1, 26-28). Furthermore, we have recently shown that systemic LPS levels are reduced but not normalized despite 2 years of ART with viral suppression (37).

In three preceeding clinical explorative trials, we have demonstrated that COX-2 inhibition by COX-2 inhibitors (COX-2i) improves the immune functions of HIV patients on ART (15, 22, 29). In the last trial (29) we also showed for the first time that treatment with a COX-2i was able to downregulate chronic immune activation and improve T cell functions (efficacy of T cell-dependent vaccine) in asymptomatic HIV-infected patients who did not use ART. In these patients, chronic immune activation was dampened as

demonstrated; CD38 density on CD8+ T cells (primary endpoint) decreased by 24% by study week 12. This reduction could be extrapolated to a possible improvement of CD4+ T cell loss with 30 CD4 cells $\mu l^{-1} year^{-1}$; i.e. a CD4 loss of 60 $\mu l^{-1} year^{-1}$ cells per year (approximately mean CD4 loss rate for chronic HIV infection) might be reduced to 30 CD4+ T cells $\mu l^{-1} year^{-1}$. These data founded the basis for further support to this study through the GLOBVAC call program under the Norwegian Research Council (granted application).

The primary goals of this study are to examine whether these observations can also be achieved (i) by another COX-2i than celecoxib (drug class effect); (ii) at more conventional doses than the preceeding study which used a celecoxib dose as high as 400 mg bid; and (iii) avoid a high frequency of rash obtained by celecoxib in contrast to what we found with identical doses in patients on ART, a rash which to our surprise was associated by high immune activation levels itself and may have represented other bioprocesses than conventional drug allergies (29). The secondary goals are to explore whether this new immunomodulating strategy could represent an affordable and simple alternative for a substantial number of HIV-infected people in resource-poor areas to slow down disease progression and/or enhance vaccination efficacy in these patients. Thereby onset of certain opportunistic infections in general may be delayed and vaccines may work more efficiently, including efficacy of therapeutic HIV vaccines. Moreover, the optimal duration of etoricoxib both in the pre- and post-vaccination period will be explored.

The widespread use of COX-2i recently revealed some drug-related increase in cardiovascular events, particularly in elderly patients at risk for such adverse events (30-32). However, this picture is less obvious in HIV-infected individuals because chronic immune activation *per se* is an important factor which contributes to the enhanced cardiovascular risk even in patients off ART (33-34). In our last study, a number of protrombotic factors and markers for endothelial damage were measured, finding only a slight increase of D-dimer in the control group and possibly a reduction of the pro-atherogenic cytokine IL-6 in the intervention group (29). However, also in this trial patients with typical high cardiovascular risk will be excluded and parameters reflecting activated coagulation and endothelial damage will again be monitored.

2 OBJECTIVES

Primary:

To explore the efficacy of etoricoxib on progression markers and vaccine responses in HIV-infected patients on effective ART with subnormal CD4 counts and off ART.

Secondary:

To explore the safety of etoricoxib in HIV-infected patients.

3 STUDY DESIGN

Etoricoxib 90 mg qd will be studied in patients with a confirmed diagnosis of HIV infection < 8 years prestudy on and off ART(http://www.bhiva.org/ClinicalGuidelines.aspx). The study will be open and randomize patients to either etoricoxib for 2 or 24 weeks or control arm.

Approximately 20-30 patients not receiving ART and 20-30 patients receiving ART will be included in one single centre in this phase IIa open explorative study. After informed consent and screening at week -12, each patient randomized to etoricoxib will go through a 2 or 24 weeks treatment period. The study design randomizing patients into three study arms in a 2:1:1 fashion will test the

-efficacy of Cox-2 inhibitor on systemic immune activation after 25 weeks (Arm 1)

-vaccination efficacy of pre-treatment with Cox-2 inhibitor for 1 week (Arm 2) or 5 weeks (Arm 1) before vaccination

-vaccination efficacy of post vaccination treatment with Cox-2 inhibitor for 1 week (Arm 2) or more than 20 weeks (Arm 1)

If intolerable adverse reactions occur at any time during the treatment period, the dose of investigational product may be reduced or stopped. A post-study drug control follow-up will end the study at week 18.

For study flow-chart, see also section 6.1.

Study week	Screening<br>-12/-2		0	1	2	3	4	5	6	7	8	9	16	17	18	24	25	36
Arcoxia24<br>(Arm #1)		-->	Salr					V<br>Salr				Ir		Salr			Salr	Salr
Arcoxia2<br>(Arm #2)		-->	Salr	V				Salr				P		Salr			Salr	Salr
Control<br>(Arm #3)		-->	Salr	V				Salr				P		Salr			Salr	Salr

Shaded areas indicate use of etoricoxib qd in patients randomized to study arms 1-3.
V, vaccination (see section 5.2.1.3 for details); Sa, safety variables; Ir, immune response assessments; P, phone interview.

SELECTION OF STUDY POPULATION

3.1 Inclusion Criteria

Subjects may be included in the study if they meet all of the following criteria:

- HIV-seropositive < 8 years and no history of symptomatic acute HIV infection within the last 12 months
- Age between 18 to 65 years
- In patients not receving ART:
  - No HIV related clinical manifistations indicating ART
  - CD4 T cell counts > 350 * 10^6/l
  - Plasma HIV RNA > 2000 copies/ml
- In patients receiving ART > 12 months:
  - Viral suppression with HIV RNA < 50 copies/ml > 6 months.
  - Current CD4+ T cell counts < 500 * 10^6/l
- Signed informed consent

3.2 Exclusion Criteria

Subjects must be excluded from participating in this study if they meet any of the following criteria:

3.2.1 Study-specific exclusion criteria

- Concomitant or sporadic use of NSAID or corticosteroids (>2 times per week)
- Other immune modulating therapies including interferon-alpha
- Total cholesterol > 7 μM
- Under treatment for hypertension or antihypertensive treatment indicated at inclusion
- Cardiovascular events or stroke in parents, siblings or off-springs occurring < 55 years of age
- Serum creatinine above reference levels (females > 90 μM; males > 100 μM), see reduced creatinine clearance in Section 4.2.2 below)
- Diabetes type I or II

3.2.2 Exclusion criteria according to the Summary of Product Characteristics (SPC) on Arcoxia from Norwegian Medicines Agency

- Known hypersensitivity for etoricoxcib or etoricoxcib capsule substances
- Known hypersensitivity for sulphonamides

- Active peptic ulcer or gastrointestinal haemorrhage
- Patients with history of asthma, acute rhinitis, nasal polyps, angioneurotic oedema, urticaria or other allergic reactions after taking acetyl salicylic acid or NSAID including COX-2 inhibitors
- Pregnancy or insufficient birth control for females
- Breastfeeding
- Seriously deranged liver function (serum albumin<25 g/l or Child-Pugh>=10).
- Patients with creatinine clearance < 30 ml/min
- Inflammatory bowel disease
- Heart failure (NYHA II-IV)
- Established ischaemic heart disease, peripheral arteriosclerosis and/or cerebrovascular disease, including previous myocardial infarction, angina pectoris, unstable angina, PCI or coronary bypass, previous transitory ischemic attack or apoplexia/stroke.

3.3 Withdrawal and Termination Criteria

In accordance with the Declaration of Helsinki, each subject is free to withdraw from the study at any time. An investigator also has the right to withdraw subjects from the study in the event of inter-current illness, adverse events or other reasons concerning the health or well being of the subject, or in the case of lack of cooperation.

Should a subject decide to withdraw, or should the investigator decide to withdraw the subject, efforts will be made to complete and report the observations up to the time of withdrawal as thoroughly as possible. A complete final evaluation at the time of the subject's withdrawal should be made and an explanation given of why the subject is withdrawing or being withdrawn from the study.

The reason, date, and time for withdrawal will be noted in the Case Record Form (CRF). If the reason for withdrawal is a clinical adverse event or an abnormal laboratory test result, monitoring will continue until the outcome is evident. If possible, measurements and recordings should continue according to the protocol.

The specific event or test result(s) being the reason for withdrawal will be recorded in the CRF.

Approximately 50-60 patients will be enrolled. Patients dropping out will not be replaced.

4 TREATMENT

4.1 Selection of Drugs and Doses

The immunomodulatory effects of Cox inhibitors were expected to be dose-dependent; hence in the preceeding trial on untreated patients (UUSCOX2, EudraCT No 2006-001882-41), the highest tolerated dose (400 mg bid, a dose approved by FDA in familial adenomatous polyposis (FAP)) were investigated in an exploratory study (29). A relatively high frequency of generalized rash occurred almost at the same day (day 12) in 4 out of 17 included patients (REF) in contrast to 0 out of 19 patients in patients taking celecoxib together with ART (REF AIDS 2006). The fact that these patients probably had higher levels of chronic immune activation than the others was suggestive for that rashes did not represent conventional allergic reactions, analogous to transient rash observed for effective ART drugs such as efavirenz.

In this study etoricoxib was selected rather than celecoxib due to the high frequency of rash observed as described above, and because this drug is once daily, which in our experience is important for patients who otherwise do not use drugs, and due to the Cox-2-specificity for etoricoxib.

In contrast to the celecoxib studies, in which doubled of general maximal doses were used, we here intend to use etoricoxib at a regular dose at 90 mg qd which is a standard dose in the long-term treatment for example of rheumatoid arthritis.

If intolerable adverse reactions occur, the dose may be reduced or stopped.

The treatment will be administered for 2 (Arm #2) or 24 weeks (Arm #1).

4.2 Treatments Administered

The following treatment will be administered:

1. Arcoxia (etoricoxib) tablets, 90 mg, to be administered in one daily dose.

4.3 Investigational Product

The investigator is responsible for ensuring that investigational product is correctly received and recorded, handled and stored and used in accordance with this protocol.

All investigational product containers (opened, unopened, or empty) must be returned to the sponsor after the study. A drug dispensing log must be prepared for each subject. At weeks 0, 6 and 12 and at any other relevant time-point the following information must be entered into the drug dispensing log:

-date of visit

-number of containers given to the patient, including individual container number(s)

-number of containers returned from the patient, including individual container number(s) and whether they are unopened, opened or empty

-the number of tablets left in the containers which are opened, but not empty

-explanation of any discrepancies

-signature of the person distributing/collecting the container(s)

After completion of the study, the completed drug dispensing logs must be signed by the investigator.

4.3.1 Supply and Packaging

Investigational product will be bought as ordinary marketed product by the Investigator and kept in stock, dispensed and labelled by the Oslo University Hospital Pharmacy Ullevål according to § 5-4 in "Forskrift om klinisk utprøving av legemidler til mennesker" av 30.10.2009" as:

Arcoxia tablets 90 mg no 200

In addition to the standard labelling, each container will comprise an individual container number and the following information:

TIL KLINISK UTPRØVING

OUSCOX2 Pasientnr *(to be filled in)*

Text: 1 tablett 1 gang daglig. Oppbevares utilgjengelig for barn

Utprøver: Professor Dag Kvale, Infeksjonsmedisinsk avdeling,
Oslo universitetssykehus HF, Kirkeveien 166, 0407 Oslo
Telefon 22119100 / 95200709. Ta kontakt i nødstilfelle for informasjon
om Arcoxia og studien.

TA MED PAKNINGEN VED NESTE KONTROLL

Ikke kast tomme pakninger

If the dose is reduced in accordance with the protocol, a sticker with the new dosage regimen will be put to cover the previous dosage regimen. The sticker will read:

1 tablett 1 gang daglig

4.4 Method of Assigning Subjects to Treatment Groups

The subjects will be included by consecutive subject numbers from 1 and upwards.

4.5 Randomization

At inclusion week 0, the patients in each stratum (on and off ART) will be consecutively randomized in groups of 20 so that all four treatment arms are equally represented at inclusion of every 10th patient. Participants will be randomized to either "Arcoxia24", "Arcoxia2", or "Control", respectively, according to Study group descriptions.

4.6 Prior and Concomitant Therapy

Any prior and concurrent therapy or medication given to a subject within 7 days before and up to the end of the observation period (week –1 to 24), will be recorded in the CRF. Trade name, dosage and indication will be recorded.

The following medication is not permitted during the study in either group:

Other NSAIDs, ASA or other immune modulating therapies.

If the patient’s clinical, immunological and/or virological situation is changed during the study, antiretroviral or other therapy will be instituted according to the investigator’s evaluation and good clinical practice.

4.7 Treatment Compliance

Compliance will be calculated based on the number of returned tablets and the number of planned days with study drug.

5 EFFICACY AND SAFETY VARIABLES

5.1 Efficacy and Safety Measurements (Study Schematic)

Weeks	-12 to -2	0 *Drug start*	1 Arms 2+3 only	5	9	17	25 *Drug stop Arm 1*	36
Informed consent	X							
Subject screening	X	X						
Demographics		X						
Medical history	X	X						
Concom. med.		X	X	X		X	X	X
Vital signs		X		X		X	X	X
Physical exam		X				X		X
Phone interview					X (Arms 2+3)			
Clinical chemistry	X[a]	X[a]		X		X	X	X
Biological markers	X[a]	X[a]		X		X	X	X
Drug dispensing		X		X (Arm 1)		X (Arm 1)		
Drug collecting				X (Arm 2)			X (Arm 1)	
Vaccination (T)			X (Arms 2+3)	X (Arm 1)				
Adverse events		X	X	X	X	X	X	X
In vitro immune parameters		X		X	X (Arm 1)	X	X	X
In vivo immune response (postvaccination)		X		X	X (Arm1)	X	X	X

Footnote: [a]Duplicate samples will not be preformed for patients with screening within one month prior to inclusion.

5.2 Efficacy Assessments

The efficacy analyses will be performed at the Department of Infectious Diseases, Oslo University Hospital, Oslo and Norwegian Institute of Public Health.

5.2.1 Efficacy Variables

Systemic activation markers at WEEKS 0, 9 and 25:

1) Plasma virus load

2) Quantification in peripheral blood of CD4+ and CD8+ T lymphocytes

3) β_2-microglobulin in plasma

4) Immunoglobulins in serum

5) CD38 expression on CD8+ T-cells

Serum antibody titres to vaccine antigens and T cell responses to vaccine antigens, HIV, and control antigens at WEEKS 0, 5, 9 and 25 weeks (see section 6.2.1.3).

5.2.1.1 Primary Efficacy Endpoints

Systemic immune activation: Changes (deltas) in CD38 expression on CD8 T-cells

Vaccine immune responses: Changes (deltas) in humoral and cellular immune responses to specific vaccine antigens

5.2.1.2 In vitro immune parameters

Several functional immune assays will test time- and drug-dependent aspects of cellular immune response to non-specific and specific stimuli. These tests will be carried out at the different partner sites with corresponding technologies to the previously published papers by the partners (see References).

5.2.1.3 Immune parameters after in vivo vaccination

To examine any effects of etoricoxib on functional immunological responses in vivo, we will in collaboration with The Norwegian Institute of Public Health (Folkehelseinstituttet) ask patients in all study arms to volunteer for vaccination, randomized to either Study weeks 1 or 6 with several standard T cell-dependent standard and approved vaccines, some which are indicated in chronic HIV infection, namely

- Seasonal influenza-vaccine (for expected outbreak)
- Tetanustoxoid (a T cell-dependent protein vaccine preventing tetanus, here mainly recall antigen in that most pts have received the vaccine before age 20)
- Hepatitis A
- Pneumococcal polysaccharide vaccine, conjugated
- Human papillomavirus (HPV, recombinant) – optional in subproject, many HIV-infected exposed to HPV

Serum antibody titres as well as T cell responsiveness in PBMC to the various antigens will be assessed before and after vaccination.

Patients who agree on vaccination, an important aspect of the main study, will sign separate Informed consents to each vaccine on same Consent form. Exposure to one of the same vaccine < 6 months earlier or previous adverse event to one or several of these vaccines render the patient unable to receive the same vaccine again, or for other reasons determined by the patients or his/her physician.

5.3 Safety Assessments

The investigator and the sponsor's medical expert will review the safety data throughout the course of the study.

The following safety variables will be evaluated at weeks -4, 0, 8 and 12:

- Adverse events (AEs)
- Laboratory variables: Hb, leucocytes, thrombocytes, CRP, Na, K, Ca, creatinine, ASAT, ALAT, LDH, bilirubin, amylase, CK, cholesterol, HDL, triglycerides.

- Negative pregnancy test (urine)
- Vital signs: Systolic/diastolic blood pressure and heart rate.
- Physical symptoms and signs
- In patients receiving ART: serum concentration of protease inhibitors and efavirenz and other antiretroviral drugs for which assays are routinely available, although no interactions have been reported to occur between these drugs and the study drug.

The routine laboratory analyses will be performed at the Central Laboratory at Oslo University Hospital.

5.3.1 Medical History

A summary of the patient's relevant medical history should be recorded on the appropriate CRF page(s). The following information should be collected:

- Diagnosis/event
- Time of diagnosis/occurrence
- Duration
- Treatment
- Outcome

5.3.2 Physical Examination

A qualified physician will conduct a physical examination.

Any pathological physical examination finding after start of treatment that is classified by the investigator as a clinically significant worsening compared to previous exam will be considered an AE, documented on the subject's CRF, and followed until the outcome is determined.

5.3.3 Vital Signs

Vital signs to be measured: Systolic/diastolic blood pressure and heart rate.

Before vital signs are measured, the subject should be resting for at least 5 minutes. The same position will be used each time vital signs are measured for a given patient.

Any vital sign value after start of treatment that is judged by the investigator as a clinically significant worsening compared to previous exam will be considered an AE, documented on the subject's CRF, and followed until the outcome is determined.

5.3.4 Clinical Laboratory Evaluation

The investigator will interpret all clinical laboratory test results, using the following categories:

0= Value within reference range

1 = Value out of reference range but not clinically significant

2 = Value out of reference range and clinically significant

Any laboratory value after start of treatment that is judged by the investigator as a clinically significant worsening compared to previous exam will be considered an AE, documented on the subject's CRF, and followed until the outcome is determined.

The signed and interpreted laboratory results will be kept together with the subject's CRF as supplemental pages.

5.3.5 Adverse Events (AEs)

The adverse drug reaction (ADR) profiles etoricoxib give reason to expect gastrointestinal ADRs as the most commonly occurring. Headache and dizziness may also be expected. In addition, study personnel must remain vigilant for the occurrence of any event related to the patients' AIDS/HIV diagnosis as well as to all other types of AEs.

An AE is defined as any unfavourable and unintended sign, symptom, or disease temporally associated with the use of a medicinal (investigational) product, whether or not considered related to the investigational product. Only symptoms/signs that begin or worsen in severity after the start of investigational product administration will be recorded as AEs in the case report form.

The patients will be closely observed and questioned for any kind of AE during the study. The patients will be instructed to immediately report any symptoms and signs to the study staff, also between the formal controls.

If an AE has already been reported it is not necessary to report each individual sign and symptom of that AE as a separate AE. For example, if Myocardial Infarction is reported as an AE there is no need to report an elevated CK, abnormal ECG or other related signs, symptoms or laboratory values as separate AEs.

5.3.6 Serious Adverse Events (SAEs)

An SAE is defined as any adverse event that:

- Resulted in death.
- Was immediately life-threatening.
- Required inpatient hospitalisation.
- Resulted in persistent or significant disability or incapacity.
- Was a congenital anomaly or birth defect.

5.3.7 Adverse Event and Serious Adverse Event Reporting

All AEs reported by the subject or observed by the hospital personnel will be reported in the CRF. The following information regarding each AE will be obtained: date and time of onset and resolution (duration), severity (defined below), whether it was serious (defined in Section 5.3.6), any required treatment or action taken, outcome, relationship to study drug, relationship to the patient's AIDS/HIV diagnosis and whether the AE caused withdrawal from the study.

All AEs should be recorded using accepted diagnoses if possible. Changes in laboratory results, vitals signs, ECGs, and physical exams should NOT be recorded as AEs if they are known to be a symptom or sign of a syndrome or diagnosis that is also being reported as an AE. For example, ST elevation on the ECG and increase in serum CK are known signs of myocardial infarction, and need not be reported if myocardial

infarction is reported as an AE. However, if both occurred in isolation and myocardial infarction was not diagnosed, then the event would be reported as an AE.

The severity of all AEs will be graded as mild, moderate, or severe using the following definitions:

Mild: Tolerable

Moderate: Interferes with normal activity

Severe: Incapacitating (causes inability to perform usual activity or work)

In addition to the investigator's own description of the AE, each AE will be encoded according to a well-recognised dictionary of medical codes.

Investigator will report SAEs to the EC (Regional komité for medisinsk forskningsetikk, Helseregion Øst, REK I) and the National Regulatory Authority (Statens Legemiddelverk), according to national regulations ("Forskrift om klinisk utprøving av legemidler til mennesker" av 30.10.2009", and "Standard prosedyrer for saksbehandling, De regionale komitéer for medisinsk forskningsetikk", revision of 8 Nov 2001).

SAE that are fatal or life-threatening will be reported within 7 days of becoming aware of the SAE. All other SAE reports are to be reported within 15 calendar days, allowing time for more complete reports. All SAE reports will be communicated electronically to Norwegian Medicines Agency preferably in an E2B compatible format as well as be recorded in the CRF and monitored until the outcome is determined. SAEs will be recorded if they occurred as follows:

- between the first administration of the study drug and the completion of the last follow-up evaluation, whether or not considered related to the investigational product

- at any time after completion of the last follow-up evaluation, and came to the investigator's attention and were judged to be related to the subject's participation in the study

6 DATA HANDLING AND QUALITY ASSURANCE

6.1 Completing, Signing and Archiving Case Report Forms (CRFs)

The investigator will keep a screening log, recording all subjects who were screened and whether they were enrolled or not. A separate Subject Identification List will be kept, showing code numbers, names, and dates of birth to allow unambiguous identification of each subject included in the study. A note will be made in the hospital medical records that the subject is participating in a clinical study.

The CRF will be completed legibly in **black ink**, with reasons given for any missing data. Any errors should be corrected by a single line strike-through, and annotated with the current date and initials of the person correcting the error. Under no circumstances should data be permanently obliterated with ink or correcting fluid.

The principal investigator will sign the last page of the CRF. Any corrections to the data will be made in a manner that does not obscure the original entry and will be dated and initialled by the investigator or assigned designee.

6.2 Clinical Data Management

The investigators will be responsible for the processing and quality control of the data.

6.3 Archiving

The following information will be retained at the investigator's site for at least 15 years after publication of the final formal study report according to § 5-3 in "Forskrift om klinisk utprøving av legemidler":

- Source documents
- CRFs
- Copies of protocols
- Protocol amendments
- Drug accountability forms
- Correspondence
- Subject identification lists
- Informed consent forms
- Copies of study reports
- The original CRFs
- All data management documentation

7 STATISTICAL METHODS AND PLANNED ANALYSIS

7.1 General Statistical Considerations

To avoid multiplicity, one primary end-point will be pre-defined for the primary analyses. All subjects will be presented in separate listings. Data from subjects screened, but not included in the study, will not be presented in any tables or listings.

7.2 Subject Characteristics

A table will be provided with the following information:

- number of subjects, both screened and enrolled, included in the study
- number of subjects included in the efficacy analysis
- number of subjects included in the safety analysis
- number of subjects withdrawn from the study and the reason for withdrawal

7.2.1 Study Population Variables

Demographic information (age, height, weight) will be summarized using descriptive statistics. Gender and race will be summarized by counts.

Medical histories will be summarized by counts. Concurrent medications will be recorded and coded using a standard classification system and grouped by primary and secondary classes if applicable.

7.3 Safety Analysis

7.3.1 Adverse Events

The number and percentages of patients with at least one AE will be tabulated. Occurrence of particular AEs and their severity and relationship to study drug will be summarized.

7.3.2 Vital Signs

Categorical data will be summarised by treatment and as a total using count and percentages of patients. Continuous data will be summarised by treatment and as a total using medians, quartiles and ranges.

7.3.3 Physical Examinations

Categorical data will be summarised by treatment and as a total using count and percentages of patients. Continuous data will be summarised by treatment and as a total using median, quartiles and ranges.

7.3.4 Clinical Laboratory Measurements

Categorical data will be summarised by treatment and as a total using count and percentages of patients. Continuous data will be summarised by treatment and as a total using median, quartiles and ranges.

7.4 Efficacy Analysis

Estimated of clinically relevant changes in efficacy parameters will at least be defined as follows:

1)	Plasma virus load	±0.5 log step
2)	CD4+ in peripheral blood	$\pm 50 \times 10^6$ /l
3)	β-2 microglobulin in plasma	±20%
4)	Immunoglobulins in serum	±20%
5)	CD38 expression on CD8+ T cells	±10%

7.5 Sample Size Determination

It is difficult to extrapolate longitudinal variation in the primary efficacy variables since data are sparse or non-existing. We have proven differences in CD38 after 12 weeks on high dose celecoxib (Pettersen et al., 2010) with 13 and 14 patients in the rx and control groups, respectively. After randomization we will aim to include minimum 15 patients in each of the study arms. A 2:1:1 randomization will secure sufficient data for long-term Arcoxia (Arm #1) which can be compared to each of the remain two, Arms #2 or #3 combined, and matched controls from our approved clinical database.

7.6 Significance Level

Groups will be compared with non-parametrical statistical methods throughout with two-ways tests using p-values equal to or below 0.05 as significant. No adjustments due to multiple comparisons will be performed.

7.7 Procedures for Missing, Unused and Spurious Data

The investigators can make the decision regarding individual values belonging to a subject to be excluded from the statistical evaluations, but only when a protocol violation is considered to weaken any of the scientific aspects of the study. Missing data will not be substituted by estimated values, but will be treated as missing data by the method used.

7.8 Rules for Excluding Subjects from Analysis

The investigators can make the decisions regarding individual values belonging to a subject to be excluded from the statistical evaluations.

7.9 Procedures for Reporting Deviations from Original Statistical Plan

Any deviations from the statistical analysis outlined in this protocol will be described, and reasons for the deviations listed, in the final Clinical Study Report.

8 SPECIAL REQUIREMENTS AND PROCEDURES

8.1 Ethics Committee Review

Before starting this study, the protocol will be submitted to the national Regulatory Authority (Statens Legemiddelverk / Norwegian Medicines Agency) and to the EC (Regional komite for medisinsk forskningsetikk, Helseregion Sør, REK II) for evaluation. The study will not start before the EC and Regulatory Authority give approval or a favourable opinion.

The investigator(s) will ensure that the conduct of the study conforms to the Declaration of Helsinki (current revision) and with national laws and regulations for clinical research ("Forskrift om klinisk utprøving av legemidler til mennesker", 01 May 2004).

8.2 Handling and storage of biological material

Blood cells and serum/plasma will be frozen for immunological analysis and implemented in an approved research biobank entitled "Forskningsbiobank Infeksjonssykdommer" at The Department of Infectious Diseases, Oslo university hospital HF at the Ullevål site. The research biobank will contain only patient codes, i.e. non-identifiable information.

8.3 Changes to the Conduct of the Study or Protocol

No changes from the final approved (signed) protocol will be initiated without the EC's and the Regulatory Authority's prior written approval or favourable opinion of a written amendment, except when necessary to eliminate immediate hazards to the subjects or when the change involves only logistics or administration. The investigator will sign the protocol amendment. Any significant deviation from the protocol when no approved amendment exists will be regarded as a protocol violation, and will be addressed as such during the reporting of the study.

Before any subjects are enrolled in the study, a list of potential protocol violations and deviations will be created, with corresponding actions to be taken.

8.4 Investigator's Responsibilities

8.4.1 Overall Responsibilities

This study will be conducted in full accordance with the current revision of the Declaration of Helsinki, the Good Clinical Practice (GCP): Consolidated Guideline approved by the International Conference on Harmonisation (ICH), and any applicable national and local laws and regulations.

The investigator(s) is responsible for performing the study in accordance with this protocol and the ICH guidelines on GCP, and for collecting, recording, and reporting the data accurately and properly. Agreement of the investigator to conduct and administer this study in accordance with the protocol will be documented in a separate study agreement.

The investigator is responsible for giving information about the study to all staff members involved in the study or in any element of subject management, both before starting the practical performance of the study and during the course of the study (e.g., when new staff become involved).

The investigator(s) is responsible for ensuring the privacy, health, and welfare of the subjects during and after the study. The investigator must be familiar with the background and requirements of the study, and with the properties of the study drugs as described in the package insert.

The investigator has the overall responsibility for the conduct and administration of the study at the centre, and for contacts with study centre management, the EC and with local authorities.

8.4.2 Subject Informed Consent

Written informed consent will be obtained from each subject before any procedures or assessments are done and after the aims, methods, anticipated benefits, and potential hazards are explained. It will also be explained to the subjects that they are free to refuse entry into the study and free to withdraw from the study at any time without prejudice to future treatment.

The subject’s willingness to participate in the study will be documented in writing in a consent form, which will be individually dated and signed by the subject and by the person conducting the informed consent process. The investigator will keep the original consent forms and copies will be offered to the patients.

Written and oral information about the study in a language understandable by the subject will be given to all subjects. The information provided must include an adequate explanation of the aims, methods, anticipated benefits, potential hazards, and insurance arrangements in force.

8.4.3 Direct Access to Source Data/Documents

Authorised personnel of the Regulatory Authority inspector(s) or their agents will be given direct access to source data and documentation (e.g., medical charts/records, laboratory results, printouts, etc.) for source data verification, provided that subject confidentiality is maintained.

8.4.4 Confidentiality Regarding Study Subjects

The investigator must assure that the privacy of the subjects, including their personal identity and all personal medical information, will be maintained at all times. In CRFs and other documents submitted to the sponsor, subjects will not be identified by their names, but by initials and allocation number.

Personal medical information may be scrutinised for the purpose of verifying data recorded in the CRF. This may be done by the regulatory authorities. Personal medical information will always be treated as confidential.

8.5 Study Monitoring

The investigators may engage an external monitor for practical purposes.

8.6 Audit and Inspection

The investigator must accept that regulatory authorities may conduct an inspection to verify compliance of the study with GCP.

9 INVESTIGATIVE AGREEMENT

9.1 Financial Disclosure

This study was made possible with the following Sponsors: **The Norwegian Research Council** through the "Global Helse" program *(Grant no 192514/S50: Improved vaccination responses and optional immunomodulating therapy by adjuvant administration of COX-2-inhibitor in HIV-infected patients)) and* **Oslo University Hospital**.

The principle investigator (or investigating institution) will certify that the investigators and/or the investigating institutions do not have

- compensation for participation in the study from study drug manufacturer
- significant payments of other sorts from study drug manufacturer

The principal investigator has no proprietary interest in the study drug. Kjetil Taskén is co-inventor on a patent application claiming use of COX2i for treatment of immunodefiency diseases via Lauras AS, a spin-off company from research at the University of Oslo through Oslo Research Park, University of Oslo. Taskén is involved in study design and data analysis, but will not have any patient contact.

9.2 Confidentiality

The information contained in this document is confidential and cannot be disclosed unless required by governmental regulation. Persons to whom any portion of the contents of this document is disclosed must be informed that the information is confidential and may not be further disclosed by them.

9.3 Insurance

The clinical study subjects are insured by membership in "Legemiddelansvarsforeningen".

10 REFERENCES

1. Douek DC, Roederer M, Koup RA. Emerging concepts in the immunopathogenesis of AIDS. Ann Rev Med. 2009;60(1):471-484.

2. Douek DC, Picker LJ, Koup RA. T cell dynamics in HIV-1 infection. Annu Rev Immunol. 2003;21:265-304.

3. Ford ES, Puronen CE, Sereti I. Immunopathogenesis of asymptomatic chronic HIV Infection: the calm before the storm. Curr Opin HIV AIDS. 2009;4(3):206-214.

4. Betts MR, Nason MC, West SM et al. HIV nonprogressors preferentially maintain highly functional HIV-specific CD8+ T cells. Blood. 2006;107(12):4781-4789.

5. Aandahl EM, Michaelsson J, Moretto WJ, Hecht FM, Nixon DF. Human CD4+ CD25+ regulatory T cells control T-cell responses to human immunodeficiency virus and cytomegalovirus antigens. J Virol. 2004;78(5):2454-2459.

6. Chase AJ, Yang HC, Zhang H, Blankson JN, Siliciano RF. Preservation of FoxP3+ Regulatory T Cells in the Peripheral Blood of Human Immunodeficiency Virus Type 1-Infected Elite Suppressors Correlates with Low CD4+ T-Cell Activation. J Virol. 2008;82(17):8307-8315.

7. Torheim EA, Ndhlovu LC, Pettersen FO et al. Interleukin-10-secreting T cells define a suppressive subset within the HIV-1-specific T-cell population. Eur J Immunol. 2009;39(5):1280-1287.

8. Holm M, Pettersen FO, Kvale D. PD-1 predicts CD4 loss rate in chronic HIV-1 infection better than HIV RNA and CD38 but not in cryopreserved samples. Curr HIV Res. 2008;6(1):49-58.

9. Kaufmann DE, Walker BD. PD-1 and CTLA-4 Inhibitory Cosignaling Pathways in HIV Infection and the Potential for Therapeutic Intervention. J Immunol. 2009;182(10):5891-5897.

10. Hazenberg MD, Otto SA, van Benthem BH et al. Persistent immune activation in HIV-1 infection is associated with progression to AIDS. AIDS. 2003;17(13):1881-1888.

11. Rodriguez B, Sethi AK, Cheruvu VK et al. Predictive value of plasma HIV RNA level on rate of CD4 T-cell decline in untreated HIV infection. JAMA. 2006;296(12):1498-1506.

12. Giorgi JV, Lyles RH, Matud JL et al. Predictive value of immunologic and virologic markers after long or short duration of HIV-1 infection. J Acquir Immune Defic Syndr. 2002;29(4):346-355.

13. Aandahl EM, Aukrust P, Skalhegg BS et al. Protein kinase A type I antagonist restores immune responses of T cells from HIV-infected patients. FASEB J. 1998;12(10):855-862.

14. Aandahl EM, Aukrust P, Muller F et al. Additive effects of IL-2 and protein kinase A type I antagonist on function of T cells from HIV-infected patients on HAART. AIDS. 1999;13(17):F109-F114.

15. Johansson CC, Bryn T, Aandahl EM et al. Treatment with type-2 selective and non-selective cyclooxygenase inhibitors improves T-cell proliferation in HIV-infected patients on highly active antiretroviral therapy. AIDS. 2004;18(6):951-952.

16. Torgersen KM, Vang T, Abrahamsen H et al. Release from tonic inhibition of T cell activation through transient displacement of C-terminal Src kinase (Csk) from lipid rafts. J Biol Chem. 2001;276(31):29313-29318.

17. Aandahl EM, Moretto WJ, Haslett PA et al. Inhibition of antigen-specific T cell proliferation and cytokine production by protein kinase A type I. J Immunol. 2002;169(2):802-808.

18. Abrahamsen H, Vang T, Tasken K. Protein kinase A intersects SRC signaling in membrane microdomains. J Biol Chem. 2003;278(19):17170-17177.

19. Vang T, Abrahamsen H, Myklebust S, Horejsi V, Tasken K. Combined spatial and enzymatic regulation of Csk by cAMP and protein kinase a inhibits T cell receptor signaling. J Biol Chem. 2003;278(20):17597-17600.

20. Yaqub S, Abrahamsen H, Zimmerman B et al. Activation of C-terminal Src kinase (Csk) by phosphorylation at serine-364 depends on the Csk-Src homology 3 domain. Biochem J. 2003;372(Pt1):271-278.

21. Mahic M, Yaqub S, Johansson CC, Tasken K, Aandahl EM. FOXP3+CD4+CD25+ adaptive regulatory T cells express cyclooxygenase-2 and suppress effector T cells by a prostaglandin E2-dependent mechanism. J Immunol. 2006;177(1):246-254.

22. Kvale D, Ormaasen V, Kran AM et al. Immune modulatory effects of cyclooxygenase type 2 inhibitors in HIV patients on combination antiretroviral treatment. AIDS. 2006;20(6):813-820.

23. Harris SG, Padilla J, Koumas L, Ray D, Phipps RP. Prostaglandins as modulators of immunity. Trends Immunol. 2002;23(3):144-150.

24. Hinz B, Brune K, Pahl A. Cyclooxygenase-2 Expression in Lipopolysaccharide-Stimulated Human Monocytes Is Modulated by Cyclic AMP, Prostaglandin E2, and Nonsteroidal Anti-inflammatory Drugs. Biochem Bioph Res Co. 2000;278(3):790-796.

25. Bryn T, Yaqub S, Mahic M et al. LPS-activated monocytes suppress T-cell immune responses and induce FOXP3+ T cells through a COX-2-PGE2-dependent mechanism. Int Immunol. 2008;20(2):235-245.

26. Brenchley JM, Price DA, Schacker TW et al. Microbial translocation is a cause of systemic immune activation in chronic HIV infection. Nat Med. 2006;12(12):1365-1371.

27. Paiardini M, Frank I, Pandrea I, Apetrei C, Silvestri G. Mucosal immune dysfunction in AIDS pathogenesis. AIDS Rev. 2008;10(1):36-46.

28. Jiang W, Lederman MM, Hunt P et al. Plasma Levels of Bacterial DNA Correlate with Immune Activation and the Magnitude of Immune Restoration in Persons with Antiretroviral-Treated HIV Infection. J Infect Dis. 2009;199(8):1177-1185.

29 Pettersen FO, EA Torheim, AEA Dahmet al. An Exploratory Trial of Cyclooxygenase Type 2 Inhibitor in HIV-1 Infection: Downregulated Immune Activation and Improved T Cell-Dependent Vaccine Responses. J Virol 2011; 85:6557-6566.

30. Solomon SD, McMurray JJ, Pfeffer MA et al. Cardiovascular risk associated with celecoxib in a clinical trial for colorectal adenoma prevention. N Engl J Med. 2005;352(11):1071-1080.

31. Bresalier RS, Sandler RS, Quan H et al. Cardiovascular events associated with rofecoxib in a colorectal adenoma chemoprevention trial. N Engl J Med. 2005;352(11):1092-1102.

32. Roumie CL, Choma NN, Kaltenbach L et al. Non-aspirin NSAIDs, cyclooxygenase-2 inhibitors and risk for cardiovascular events-stroke, acute myocardial infarction, and death from coronary heart disease. Pharmacoepidem Dr S. 2009;18(11):1053-1063.

33. Kuller LH, Tracy R, Belloso W et al. Inflammatory and coagulation biomarkers and mortality in patients with HIV infection. PLoS Med. 2008;5(10):1496-1508.

34. Rodger A, Fox Z, Lundgren J et al. Activation and Coagulation Biomarkers Are Independent Predictors of the Development of Opportunistic Disease in Patients with HIV Infection. J Infect Dis. 2009;200(6):973-983.

35. Hunt PW, Martin JN, Sinclair et al. T Cell Activation Is Associated with Lower CD4+ T Cell Gains in Human Immunodeficiency Virus–Infected Patients with Sustained Viral Suppression during Antiretroviral Therapy. J Infect Dis 2003; 187:1534-1543.

36. Hunt PW, Cao HL, Muzoora C et al. Impact of CD8+ T-cell activation on CD4+ T-cell recovery and mortality in HIV-infected Ugandans initiating antiretroviral therapy. AIDS 2011; 25:2123-2131.

37. Trøseid M, Nowak P, Nyström et al. Elevated plasma levels of lipopolysaccharide and high mobility group box-1 protein are associated with high viral load in HIV-1 infection: reduction by 2-year antiretroviral therapy. AIDS 2010; 24:1733-1737.
