# Supplementary material for: Immune activation and HIV-specific T cell responses are modulated by a cyclooxygenase-2 inhibitor in untreated HIV-infected individuals: An exploratory clinical trial
Source: PLoS One. 2017 May 2;12(5):e0176527. doi: 10.1371/journal.pone.0176527 (PMC5413033; doi:10.1371/journal.pone.0176527)
Supplement: S1 Table — (DOCX) [file pone.0176527.s004.docx]

**S4. Changes in immunological variables from baseline to four months, by ART use and treatment arm**

|  | **ART-naïve** | | | | **ART-treated** | | | |
| --- | --- | --- | --- | --- | --- | --- | --- | --- |
|  | **Arm 1**  **COX-2i 4 months** | **Cox-2i 2 weeks** | **No Cox-2i** | **Cox-2i 2 weeks/ No Cox-2i** | **Arm 1**  **COX-2i 4 months** | **Cox-2i 2 weeks** | **No Cox-2i** | **Cox-2i 2 weeks/ No Cox-2i** |
| **CD38 density on CD8+ T cells (molecules/cell)** | -57.53 (-657.17 - -5.41) | 90.82 (32.54 - 194.58) | 141.44 (22.23 - 252.42) | 117.90 (22.23 - 252.42) | 18.43 (-90.50 - 163.92) | 353.19 (-816.54 - 450.12) | -56.01 (-572.78 - -13.57) | -13.57 (-572.78 - 353.19) |
| **CD38 expression on CD8+ T cells (%)** | -1.05 (-7.00 - 8.70) | 2.20 (-3.10 - 6.90) | 6.20 (-0.70 - 8.10) | 6.00 (-1.60 - 7.80) | -0.80 (-4.95 - 1.50) | 3.23 (-3.40 - 4.78) | -3.35 (-12.85 - 1.70) | -0.15 (-6.60 - 4.78) |
| **CD38 density on CD8+PD-1+ T cells (molecules/cell)** | -179.26 (-982.65 - -71.47) | 307.59 (143.73 - 606.38) | 303.82 (193.40 - 378.96) | 303.82 (193.40 - 378.96) | -27.66 (-150.08 - 261.01) | 187.12 (-2 678.33 - 700.96) | 226.91 (-817.63 - 558.14) | 187.12 (-817.63 - 558.14) |
| **CD38 density on CD8+HLA-DR+ T cells (molecules/cell)** | -191.44 (-1 068.01 - 352.02) | 1 040.75 (387.59 - 1 578.73) | 66.96 (-40.32 - 356.73) | 356.73 (-40.32 - 1 127.60) | 189.94 (-256.37 - 878.23) | 480.15 (-4 665.26 - 1 855.04) | 165.46 (-1 176.36 - 746.60) | 384.19 (-1 176.36 - 842.57) |
| **PD-1 expression on CD8+ T cells (%)** | 0.55 (-2.10 - 4.80) | -1.15 (-7.40 - 3.35) | -1.70 (-2.00 - 4.30) | -1.70 (-3.00 - 4.30) | 0.00 (-3.30 - 2.70) | 0.55 (-2.80 - 2.28) | -2.95 (-3.73 - -2.30) | -2.30 (-3.73 - 0.55) |
| **ETP (%)** | -6.82 (-10.97 - 0.53) | 3.84 (1.29 - 10.44) | 11.07 (3.54 - 27.64) | 7.14 (2.42 - 16.13) | 7.30 (0.86 - 15.93) | 9.07 (6.00 - 12.76) | 4.51 (0.37 - 5.70) | 5.74 (4.51 - 9.07) |
| **Peak (%)** | -10.19 (-17.11 - 0.82) | 7.78 (-11.65 - 37.70) | 12.51 (0.23 - 75.57) | 10.15 (-5.71 - 47.48) | 17.85 (5.37 - 25.52) | 21.82 (8.89 - 40.84) | 9.19 (-13.10 - 23.79) | 16.05 (2.02 - 29.56) |
| **Plasma free TFPI (ng/mL)** | 0.36 (-1.38 - 1.78) | -0.82 (-1.38 - 1.48) | -0.89 (-1.18 - -0.76) | -0.85 (-1.28 - 0.36) | 1.17 (-0.02 - 2.03) | -1.08 (-3.09 - -0.13) | 1.53 (-15.01 - 3.21) | -0.13 (-3.09 - 1.53) |
| **Plasma free Protein S (%)** | -0.29 (-5.62 - 8.48) | -3.94 (-15.84 - -2.58) | 9.21 (1.47 - 13.66) | -0.55 (-9.89 - 6.80) | 4.02 (-1.27 - 12.59) | -1.63 (-7.19 - 4.14) | 2.05 (-1.74 - 11.31) | -0.75 (-2.62 - 6.94) |
| **Plasma D-dimer (ng/mL)** | 1.80 (-58.90 - 82.05) | 170.90 (24.30 - 196.95) | -5.30 (-11.20 - 85.40) | 54.85 (-2.35 - 183.93) | 1.00 (-18.50 - 51.25) | 33.08 (22.73 - 86.20) | -0.05 (-229.63 - 51.80) | 22.73 (-0.05 - 65.50) |
| **Plasma sCD25 (pg/mL)** | -24.40 (-110.78 - 34.52) | -45.77 (-185.07 - 254.27) | -86.71 (-242.52 - 34.63) | -86.71 (-242.52 - 34.63) | -19.72 (-55.99 - 36.29) | -88.31 (-149.17 - 32.13) | -21.08 (-51.25 - 49.78) | -35.84 (-103.72 - 46.89) |
| **Plasma sCD163 (ng/mL)** | 21.38 (-13.24 - 216.95) | 94.98 (-62.76 - 277.75) | 48.92 (44.10 - 139.45) | 50.55 (44.10 - 139.45) | -26.26 (-101.69 - 33.10) | 22.14 (-37.01 - 137.61) | 28.49 (-128.52 - 199.48) | 22.14 (-77.22 - 184.17) |
| **Plasma sCD14 (ng/mL)** | 136.30 (-1 970.50 - 4 558.16) | 1 805.43 (454.45 - 6 418.71) | 5 036.29 (2 590.54 - 5 911.98) | 2 590.54 (1 589.55 - 5 911.98) | -205.48 (-2 756.87 - 1 118.88) | 486.47 (-4 226.52 - 2 147.96) | 389.53 (-1 510.69 - 2 626.28) | 486.47 (-1 510.69 - 2 315.11) |
| **Plasma IL-6 (pg/mL)** | 0.32 (-0.06 - 1.42) | -0.26 (-0.66 - 2.07) | -0.15 (-1.16 - 0.21) | -0.15 (-0.77 - 0.21) | -0.16 (-0.80 - 0.16) | -0.14 (-0.21 - -0.01) | 0.00 (-0.44 - 0.38) | -0.14 (-0.32 - 0.24) |
| **Plasma IP10 (pg/mL)** | -35.03 (-73.27 - 31.79) | 20.70 (-69.79 - 66.69) | -16.69 (-40.62 - 29.66) | -0.36 (-40.62 - 41.76) | -2.04 (-10.67 - 13.61) | -15.00 (-39.49 - -7.26) | -0.85 (-9.67 - 81.20) | -7.79 (-16.87 - -0.33) |
| **Plasma CRP (μg/mL)** | 0.20 (-23.90 - 12.08) | 9.51 (-8.02 - 25.58) | 0.86 (-0.79 - 7.07) | 3.96 (-0.79 - 9.51) | 0.15 (-1.85 - 16.41) | -0.59 (-8.28 - 0.28) | -6.67 (-38.68 - 25.94) | -0.59 (-19.01 - 4.93) |
| **Plasma kynurenine (μmol/L)** | -0.17 (-0.44 - 0.06) | 0.26 (0.08 - 0.46) | 0.04 (-0.23 - 0.60) | 0.18 (-0.03 - 0.57) | 0.09 (-0.24 - 0.29) | 0.12 (-0.22 - 0.48) | -0.22 (-0.32 - -0.06) | -0.06 (-0.32 - 0.12) |
| **Plasma tryptophan (μmol/L)** | -2.20 (-16.55 - 5.00) | -7.85 (-11.90 - 4.55) | 7.20 (-29.40 - 14.50) | -6.20 (-14.30 - 14.50) | -0.60 (-9.00 - 12.10) | 5.35 (-3.25 - 15.45) | -8.60 (-11.80 - 9.65) | -3.25 (-8.60 - 15.45) |
| **Plasma K/T ratio** | 0.92 (0.86 - 1.12) | 1.27 (1.01 - 1.38) | 1.06 (0.89 - 1.40) | 1.27 (0.89 - 1.40) | 1.10 (0.85 - 1.17) | 0.95 (0.85 - 1.04) | 1.01 (0.79 - 1.12) | 0.98 (0.85 - 1.07) |
| **Total Gag-specific CD8 T cell response (%)** | 0.35 (-2.33 - 1.24) | -0.74 (-2.07 - 0.62) | -0.13 (-4.65 - -0.07) | -0.13 (-2.30 - -0.07) | -0.06 (-0.30 - 0.29) | 0.43 (-0.42 – 3.97) | -0.16 (-1.50 - 0.55) | 0.18 (-0.61 - 0.75) |

Data given as median and lower/upper quartile
